# Counterion Dynamics in Chiral Supramolecular Polymerization

**DOI:** 10.1002/anie.1807439

**Published:** 2026-07-31

**Authors:** Camila Montañez‐Moyano, Timo Krüger, Alejandro Martínez‐Manjarrés, Gustavo Fernández

**Affiliations:** ^1^ Universität Münster Organisch‐Chemisches Institut Münster Germany

**Keywords:** chirality, counterion, self‐assembly, supramolecular polymers, π‐conjugated systems

## Abstract

Counterions have been studied in a broad range of chemical and biological systems, and their effects are typically evaluated by comparing different ionic species. However, the dynamic effects of a single counterion within a given system remain poorly understood. In this study, we demonstrate that counterion dynamics governs assembly pathways and chirality in supramolecular polymerization. To investigate this phenomenon, we designed dicationic tetrapyridyl Pd(II) complexes bearing two BF_4_^−^ counterions, amide groups for hydrogen bonding, and either *S*‐ or *R*‐chiral side chains, and studied their supramolecular polymerization in nonpolar media. Under kinetic conditions, the *S*‐chiral complex initially self‐assembles into nanoparticle‐like metastable aggregates **(AggI)**, which over time evolve into thermodynamically controlled supramolecular polymers **(AggII)** with inverted chiroptical signal through a competitive pathway. Spectroscopic, microscopic, and computational studies indicate that subtle changes in the position of the BF_4_^−^ counterions modulate the packing of the π‐extended pyridine ligands and the hydrogen‐bonding arrangement, giving rise to distinct asymmetry amplification behavior and supramolecular chirality. These findings demonstrate that a single counterion can direct pathway‐dependent supramolecular polymerization into distinct helical states, highlighting the crucial role of charge desolvation and dynamics in governing supramolecular self‐assembly.

## Graphical abstract

A single counterion acts as a dynamic structural element directing pathway‐dependent supramolecular polymerization. Subtle counterion repositioning governs molecular packing, aggregate morphology, supramolecular chirality, and asymmetry amplification.

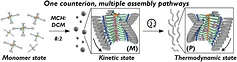

## 1 Introduction

Counterions are ubiquitous components of charged chemical and biological systems and play key roles in numerous biological functions as well as in diverse areas of chemistry, including molecular recognition [1, 2], self‐assembly [3, 4, 5], catalysis [6, 7, 8], and drug formulation [9, 10]. In supramolecular chemistry, counterions can profoundly influence the self‐assembly of charged molecular systems by modulating intermolecular interactions, molecular packing, and aggregation behavior through electrostatic, steric, and coordination effects [5, 11, 12, 13, 14, 15, 16]. This is particularly relevant in coordination compounds, where counterions may not only affect solubility and stability but also modulate intermolecular interactions that dictate supramolecular packing [17]. In particular, their size, coordinating ability, and spatial distribution can influence the relative arrangement of the ligands and thereby alter the balance between noncovalent interactions such as π−π stacking and hydrogen bonding [15, 16]. For example, variations in counterion identity have been shown to influence ligand organization and control the extent of metal–metal interactions in assemblies of coordination compounds [14]. Despite these advances, the dynamic role of individual counterions within supramolecular assemblies remains poorly understood.

Among the various supramolecular properties governed by molecular packing, supramolecular chirality is particularly sensitive to subtle structural perturbations because chiroptical properties emerge from the collective organization of molecular building blocks [18, 19, 20, 21, 22, 23, 24, 25, 26, 27, 28, 29, 30, 31]. Consequently, supramolecular polymerization often proceeds through complex energy landscapes in which multiple assembly pathways coexist, leading to kinetically and thermodynamically controlled aggregates that differ in morphology, packing modes, and supramolecular properties [32, 33, 34, 35, 36, 37, 38, 39, 40, 41]. A particularly intriguing consequence of such pathway‐dependent assembly in chiral systems is the emergence of distinct chiroptical responses, including systems displaying opposite helical senses [42, 43, 44]. Such behavior has been observed upon variations in solvent composition [45, 46, 47], temperature [44, 48], time [49, 50], light [51, 52], and other chemical stimuli that perturb intermolecular interactions within the assembly [53, 54, 55, 56]. These observations highlight the sensitivity of supramolecular helicity to subtle variations in molecular packing, underscoring the importance of controlling chirality in the design of functional supramolecular materials. Despite the recognized influence of external stimuli on supramolecular polymerization, it remains unclear whether a single counterion can control the emergence of multiple distinct aggregation pathways.

In this study, we demonstrate that counterion dynamics governs assembly pathways and chirality in supramolecular polymerization. To this end, we have designed (*S*) and (*R*)‐chiral dicationic tetrapyridyl Pd(II) complexes bearing two BF_4_^−^ counterions (**(*S*)‐1** and **(*R*)‐1**), together with a reference achiral compound **2** (Scheme 1, for synthesis and characterization, see the supporting information). In contrast to previous systems developed in our group, which relied on neutral metal complexes [57, 58, 59, 60, 61, 62, 63], the present molecular design introduces a charged architecture that enables the explicit investigation of counterion dynamics. The cross‐shaped, π‐extended geometry of the complex and the incorporation of amide groups were deliberately chosen to promote supramolecular polymerization through π–π stacking and directional hydrogen bonding interactions. Chiral side chains were introduced to induce supramolecular helicity and to allow the investigation of asymmetry transfer and amplification processes. BF_4_^−^ was selected as a small, weakly coordinating counterion because its low coordination propensity allows it to remain as a dynamic ion pair in close proximity to the Pd(II) center without perturbing its primary coordination sphere [64]. Previous studies have shown that weakly coordinating counterions can strongly influence the supramolecular organization of coordination compounds [17], making BF_4_^−^ an ideal candidate to investigate whether the repositioning of a single counterion can direct pathway‐dependent supramolecular polymerization. The chosen molecular design along with the sample processing allows the target system **(*S*)‐1** to access two competing assembly pathways with distinct morphology and supramolecular chirality in dichloromethane (DCM):methylcyclohexane (MCH) mixtures, strongly influenced by the counterion positioning. Upon solvophobic quenching, **(*S*)‐1** initially forms metastable nanoparticle‐like aggregates (**AggI**) that subsequently convert over time and/or upon heating to thermodynamically favored supramolecular polymers (**AggII**) with inverted chiroptical signal. Spectroscopic, microscopic, and computational studies reveal that the two aggregates differ in supramolecular packing, helical sense, morphology, and amplification of asymmetry, which can be traced back to the repositioning of the counterions in the course of this transformation. Our findings establish counterion positioning and dynamics as a key parameter in supramolecular polymerization, paving the way toward adaptive and chiral assemblies based on coordination compounds.

**SCHEME 1:**
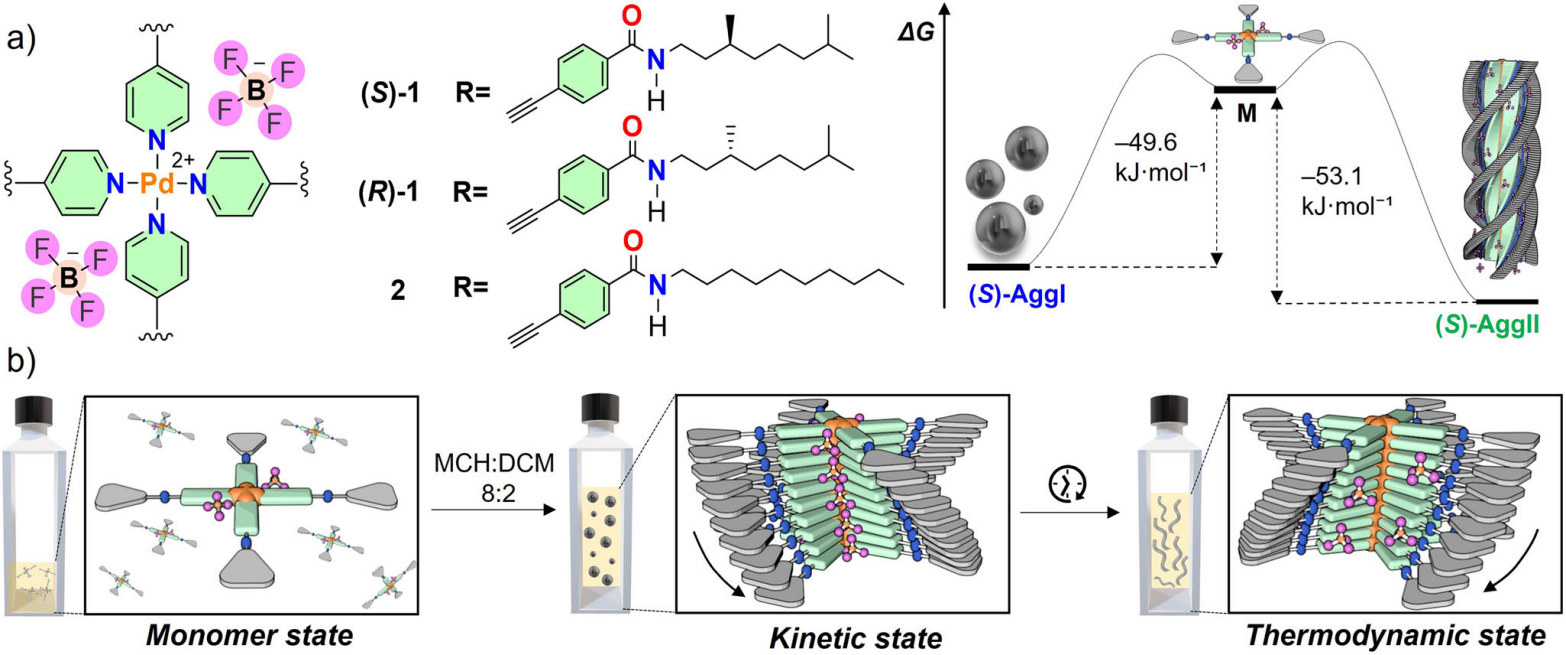
(a) Molecular structures of chiral complexes **(*S*)/(*R*)‐1** and achiral compound **2** and schematic energy landscape illustrating pathway‐dependent self‐assembly. (b) Representation of the supramolecular assembly pathway from the monomeric state to the kinetically controlled nanoparticle‐like aggregate (**AggI**) and its subsequent transformation into the thermodynamically favored fibrous aggregate (**AggII**) accompanied by helicity inversion.

## 2 Results and Discussion

Initial spectroscopic studies (Figure S17) reveal that the achiral Pd(II) complex **2** (*c* = 20 µM, 298 K) is molecularly dissolved in common organic solvents such as DCM, and chloroform (CHCl_3_), whereas aggregation occurs in nonpolar media such as MCH and *n*‐hexane (*n*‐Hex). However, due to the pronounced decrease in absorption in pristine apolar solvents arising from limited solubility, mixtures of MCH with DCM were employed to balance aggregation and colloidal stability (see Figure S16 for sample preparation). This solvent optimization provided the basis to investigate the self‐assembly of the chiral complexes **(*S*)‐1** and **(*R*)‐1** (Figures 1 and S18). In the main text, we focus on compound **(*S*)‐1**, while the corresponding data for **(*R*)‐1**, exhibiting mirror‐image CD responses, are provided in the Supporting Information (Figure S18).

**FIGURE 1:**
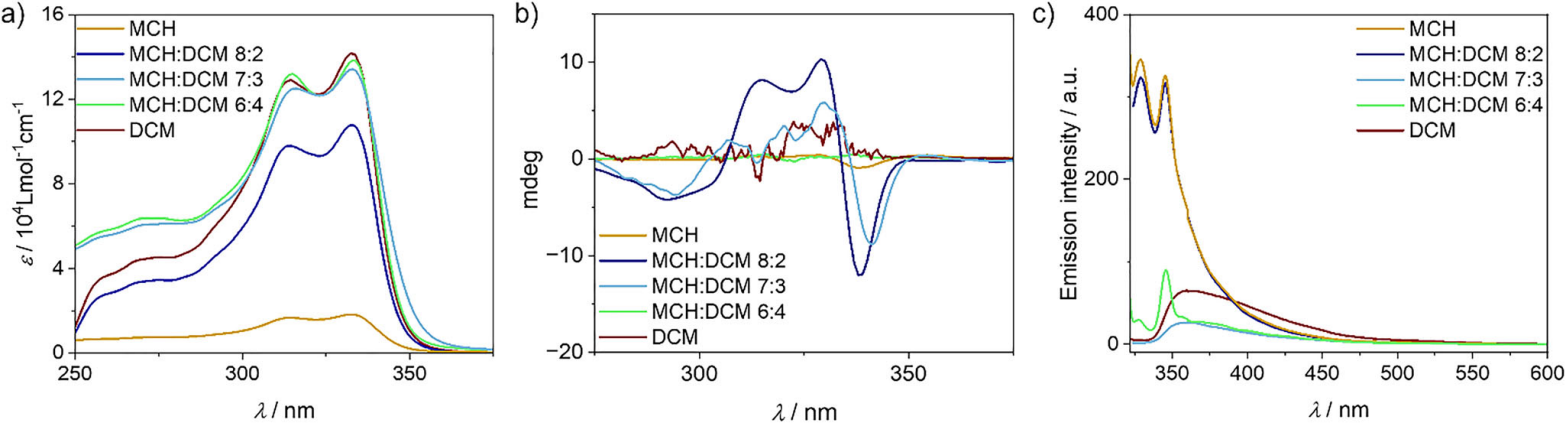
(a) absorption, (b) CD, and (c) emission spectra of **(*S*)‐1** (*c* = 20 µM, 298 K) in different MCH:DCM ratios.

In DCM (*c* = 20 µM, 298 K), **(*S*)‐1** is present in its monomeric form, as evidenced by well‐defined, intense absorption maxima at *λ *= 314 and 332 nm (Figure 1a), assigned to ligand‐centered (LC) and metal‐to‐ligand charge transfer (MLCT) transitions [65]. Under these conditions, the CD spectrum is silent (Figure 1b), and the emission spectrum shows a weak, broad band at 360 nm (Figure 1c). In contrast, in pure MCH, the limited solubility of aggregated **(*S*)‐1** leads to a pronounced decrease in absorbance (Figure 1a), in analogy to achiral **2**.

Increasing the volume fraction of DCM to 20 vol‐% colloidally stabilizes the aggregated sample and results in a minor reduction of the absorbance compared to the monomeric sample while preserving the spectral signatures (Figure 1a). The corresponding CD spectrum exhibits a bisignate (−/+) signal, with a negative band centered at 339 nm and positive bands at 314 and 328 nm (Figure 1b), indicating the transfer of asymmetry from the stereogenic centers to the supramolecular stacks during aggregation. Notably, this sample displays aggregation‐induced emission (AIE) [66]. No low‐energy emission band associated with metal‐metal‐to‐ligand charge transfer (MMLCT) transitions is observed in the 500–800 nm region (Figure 1c). This absence can be ascribed to the orthogonal arrangement of the pyridyl ligands around the Pd(II) center, which disfavors effective metal–metal interactions [67, 68]. Instead, the observed AIE is most likely associated with aggregation‐induced rigidification of the π‐extended aromatic framework. Specifically, π−π stacking and hydrogen bonding interactions restrict intramolecular motions of the π‐extended pyridine ligands, thereby suppressing nonradiative decay pathways in accordance with the restriction of intramolecular motion (RIM) mechanism [69]. Further increasing the DCM volume fraction to 30 vol‐% (MCH:DCM 7:3) results in a similar but less intense CD pattern, indicating that the aggregated state persists but with reduced stability, as supported by the increased absorbance in the UV‐Vis spectra (Figure 1a). At a DCM volume fraction of 40 vol‐%, the CD signal disappears and the absorption profile closely resembles that in pure DCM, indicating a shift toward the molecularly dissolved state. Therefore, the MCH:DCM (8:2) mixture was selected as the optimal solvent system to investigate the self‐assembly of **(*S*)‐1** and **(*R*)‐1**.

To gain further insights into the self‐assembly process, variable‐temperature (VT)‐CD and UV–Vis experiments were performed (Figures 2a–d and S19). The aggregate sample of **(*S*)‐1** (MCH:DCM (8:2), *c* = 20 µM) was initially prepared at 278 K using a solvophobic quenching approach, which favors a kinetically controlled self‐assembly (Figure S16) [36, 70]. Under these conditions, a bisignate CD band nearly identical to that observed at 298 K is obtained, confirming the formation of the same chiral aggregated species (from now on termed **(*S*)‐AggI**, Figure 2a). Interestingly, heating this solution from 278 to 323 K does not lead to dissociation of **(*S*)‐AggI** into the monomeric state, which would be accompanied by an increase in absorption. Instead, a decrease in absorbance is observed, along with changes in the relative intensities of the two maxima at *λ* = 314 and 333 nm and enhanced optical density in the 350‐400 nm region (Figure S19). These changes indicate a thermally induced reorganization into a second assembled state, denoted **(*S*)‐AggII**, which is further supported by the pronounced inversion of the chiroptical signal observed in the corresponding VT‐CD spectra upon heating from 278 to 323 K (Figure 2a). The same temperature‐dependent stereomutation was observed in dried solvents (MCH/DCM (8:2)), excluding residual water as the origin of the process (Figure S19c,d) [71, 72]. Even upon prolonged equilibration at this temperature, complete disassembly is not observed (Figure 2a), precluding thermodynamic analysis by VT experiments. Cooling the sample back to 273 K produces essentially the same spectral profile, apart from a slight increase in CD intensity (Figure 2d). The same behavior is observed for the enantiomer **(*R*)‐1**, yielding the corresponding aggregate **(*R*)‐AggII** (Figure S20). A comparable temperature‐dependent reorganization is also observed for the achiral complex **2** using VT‐UV‐Vis experiments (Figure S21).

**FIGURE 2:**
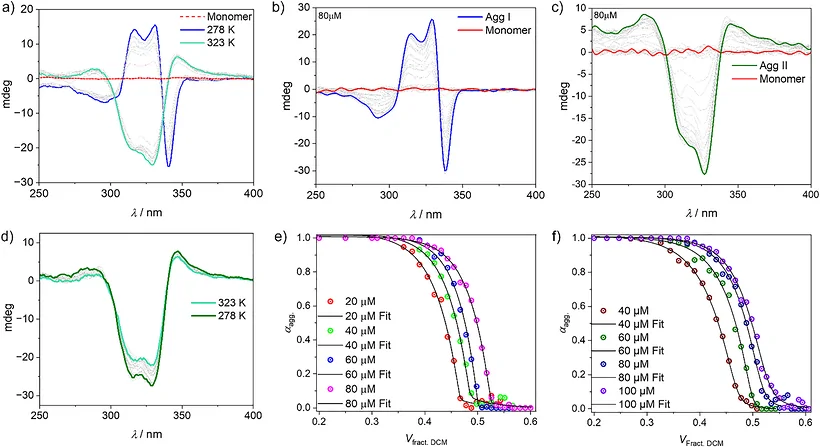
(a,d) VT‐CD experiments of **(*S*)‐1** (MCH:DCM (8:2), *c* = 20 µM): Heating (a) and cooling (d) using a rate of 1 K min^−1^. (b,c) CD denaturation studies of **(*S*)‐AggI** (b) and **(*S*)‐AggII** (c) at 80 µM and 298 K by gradually increasing the DCM (good solvent) volume fraction in MCH (poor solvent), monitoring the disassembly of the aggregates into the molecularly dissolved state. (e,f) Secondary plots derived from the CD denaturation studies at *λ* = 339 nm for **(*S*)‐AggI** (e) and **(*S*)‐AggII** (f) at 298 K in MCH:DCM mixtures at different concentrations, including fits to the denaturation model.

To compare the stability of the two aggregated states, CD solvent‐denaturation experiments were performed for **(*S*)‐AggI** and **(*S*)‐AggII** at four different concentrations (Figure 2b,c; Figures S23 and S24). In both cases, direct disassembly into the monomeric state is observed without detectable intermediates (Figure 2b,c). For **(*S*)‐AggI**, complete disassembly occurs at approximately 40 vol‐% DCM (Figure 2e). A similar solvent range is required to fully disassemble **(*S*)‐AggII** after equilibration (Figure 2f). Analysis of the resulting denaturation curves indicates that both systems follow a cooperative aggregation mechanism (Table S1). The average Gibbs free energy of **(*S*)‐AggI** (Δ*G*° = –49.6 kJ·mol^−^^1^) is slightly higher (less negative) than that of **(*S*)‐AggII** (Δ*G*° = –53.1 kJ·mol^−^^1^), indicating that **(*S*)‐AggII** is the themodynamically more stable species.

The transformation of **(*S*)‐AggI** to **(*S*)‐AggII** in MCH:DCM (8:2) was examined by time‐dependent CD measurements at varying concentrations and temperatures (Figure 3a–c; Figures S25 and S26). Over time, the initial CD pattern of the kinetic species **(*S*)‐AggI** gradually evolves into the bisignate CD band characteristic of **(*S*)‐AggII**, demonstrating a time‐dependent structural reorganization of the supramolecular assembly (Figure 3a). Increasing concentration from 30 to 500 µM significantly slows down the **(*S*)‐AggI**→**(*S*)‐AggII** conversion (Figure 3b), indicating that both aggregated states arise from competing assembly pathways. Additional kinetic studies at constant concentration and varying temperatures reveal a clear acceleration of the process with increasing temperature (Figure 3c), indicating that the **(*S*)‐AggI**→**(*S*)‐AggII** transition is thermally activated. An analogous time‐dependent behavior is observed for the enantiomeric complex **(*R*)‐1**, which undergoes the same **(*R*)‐AggI**→**(*R*)‐AggII** transformation under comparable conditions (Figure S27).

**FIGURE 3:**
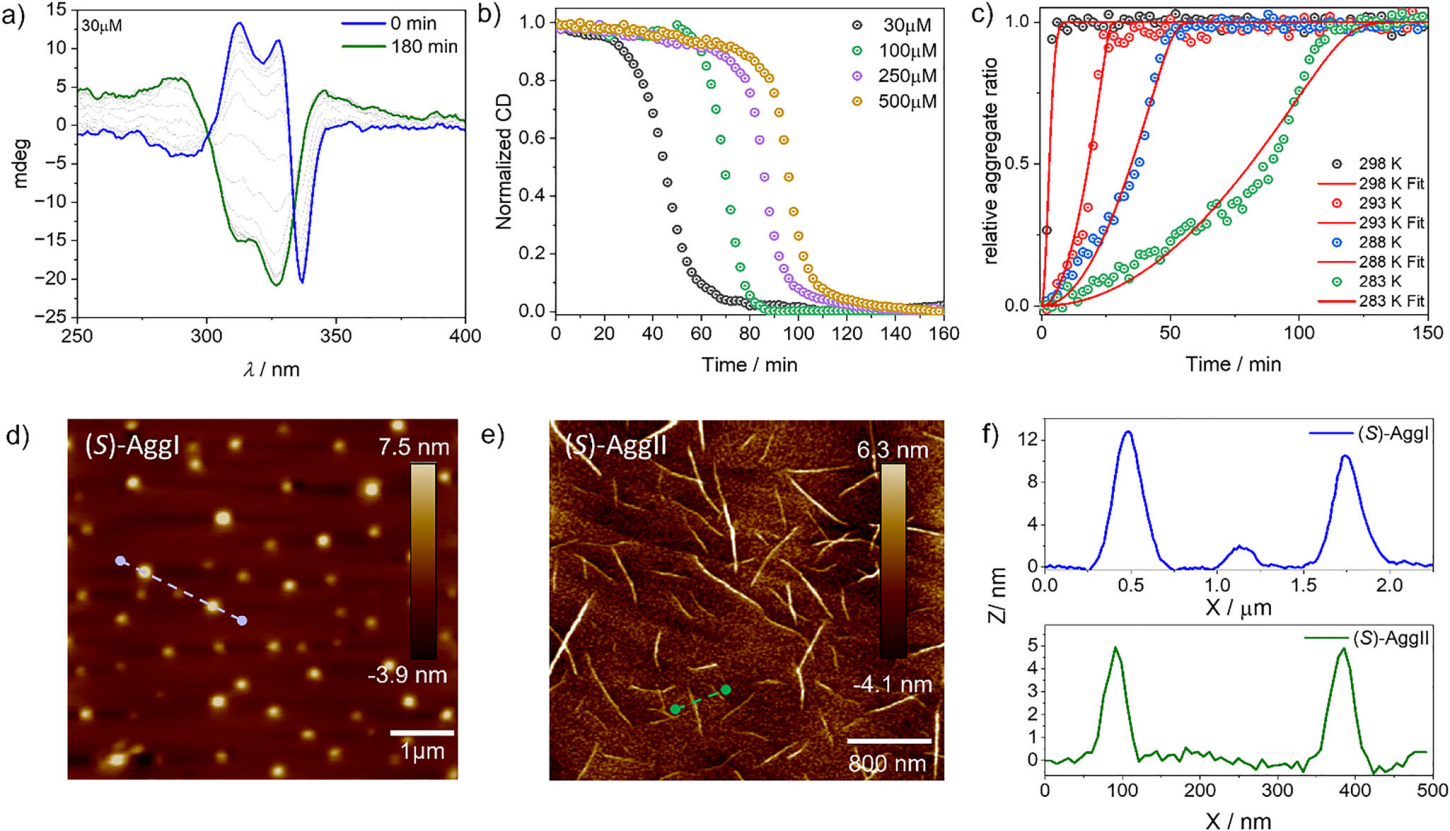
(a) Time‐dependent CD spectra showing the **(*S*)‐AggI→(*S*)‐AggII** transformation in MCH: DCM (8:2) (*c* = 30 µM, *T* = 278 K). Kinetic profiles revealing the **(*S*)‐AggI→(*S*)‐AggII** transformation solution over time (*λ* = 333 nm) at different concentrations (b) and temperatures (c). AFM height images of **(*S*)‐AggI** (d) and **(*S*)‐AggII** (e) along with height profiles (f) along the colored lines in (d) and (e).

To quantitatively describe the transformation kinetics, the time‐dependent CD traces recorded at 30 µM were fitted using an unseeded nucleation–elongation model implemented in the AmyloFit platform (Figure S28; Tables S2 and S3) [73]. This model, originally developed to describe structural transitions in protein aggregates, is well suited to monitor the kinetic profiles associated with the **(*S*)‐AggI→(*S*)‐AggII** transformation. The extracted rate constants display a clear temperature dependence, further supporting a thermally activated reorganization process. Although both aggregates exhibit similar packing motifs, their formation is governed by distinct kinetic and thermodynamic regimes, with **(*S*)‐AggI** representing a metastable intermediate that undergoes time‐ and temperature‐dependent helical inversion toward the thermodynamically preferred **(*S*)‐AggII** state.

The morphology of the two aggregated states was investigated by atomic force microscopy (AFM) on mica substrates and scanning electron microscopy (SEM) on silicon wafers. AFM images of the kinetic species **(*S*)‐AggI** (Figures 3d and S29) reveal nanoparticle‐like structures with diameters ranging from 200 to 400 nm. Height analysis indicates particles of approximately 14 nm (Figure 3f, top), which likely result from partial flattening of the particles on the surface during the drying process [74, 75]. In sharp contrast, AFM images of **(*S*)‐AggII** (Figures 3e and S30) show fiber‐like structures with lengths of several hundreds nanometers. Height profile analysis indicates fiber heights of approximately 5 nm (Figure 3f, bottom), consistent with one‐dimensional (1D) supramolecular polymers of single‐molecule width (vide infra). Similar morphological features were also observed by SEM upon solvent evaporation (Figures S31 and S32), confirming the presence of nanoparticle structures for **(*S*)‐AggI** and more ordered fibers for **(*S*)‐AggII**. The formation of particle‐like morphologies for **(*S*)‐AggI** and anisotropic assemblies for **(*S*)‐AggII** is further supported by angular‐dependent dynamic light scattering (DLS) (Figure S33).

So far, experimental evidence has revealed the existence of two competing aggregation pathways for **(*S*)‐1** and **(*R*)‐1**, leading to supramolecular assemblies exhibiting opposite chiroptical signals. However, the molecular origin of this behavior has yet to be fully elucidated. Given that both aggregates are formed from the same molecular building block and in the same solvent mixture and concentration, the observed differences in supramolecular chirality are likely to arise from subtle variations in molecular packing. Considering the known ability of counterions to modulate intermolecular interactions in charged assemblies, we hypothesized that the BF_4_^−^ counterions may play a key role in directing the formation of the two chiral aggregates. To investigate the possible involvement of the counterions during aggregation, a combination of 1D and 2D NMR experiments, including ^1^H–^1^H ROESY and ^1^H–^1^ ^9^F HOESY measurements, was performed (Figure 4a,b; Figures S34b,c and S35b). Initial solvent‐dependent ^1^H NMR experiments recorded in various CD_2_Cl_2_:MCH‐d_14_ mixtures at a concentration of 0.5 mM and 298 K reveal signal broadening and chemical shift changes upon increasing the fraction of MCH‐d_14_, consistent with the initial stages of an aggregation process (Figure S34b). In parallel, ^1^ ^9^F NMR measurements show a progressive downfield shift and broadening of the BF_4_^−^ resonances, indicating that the counterions undergo changes in their local environment under these conditions (Figure S34c).

**FIGURE 4:**
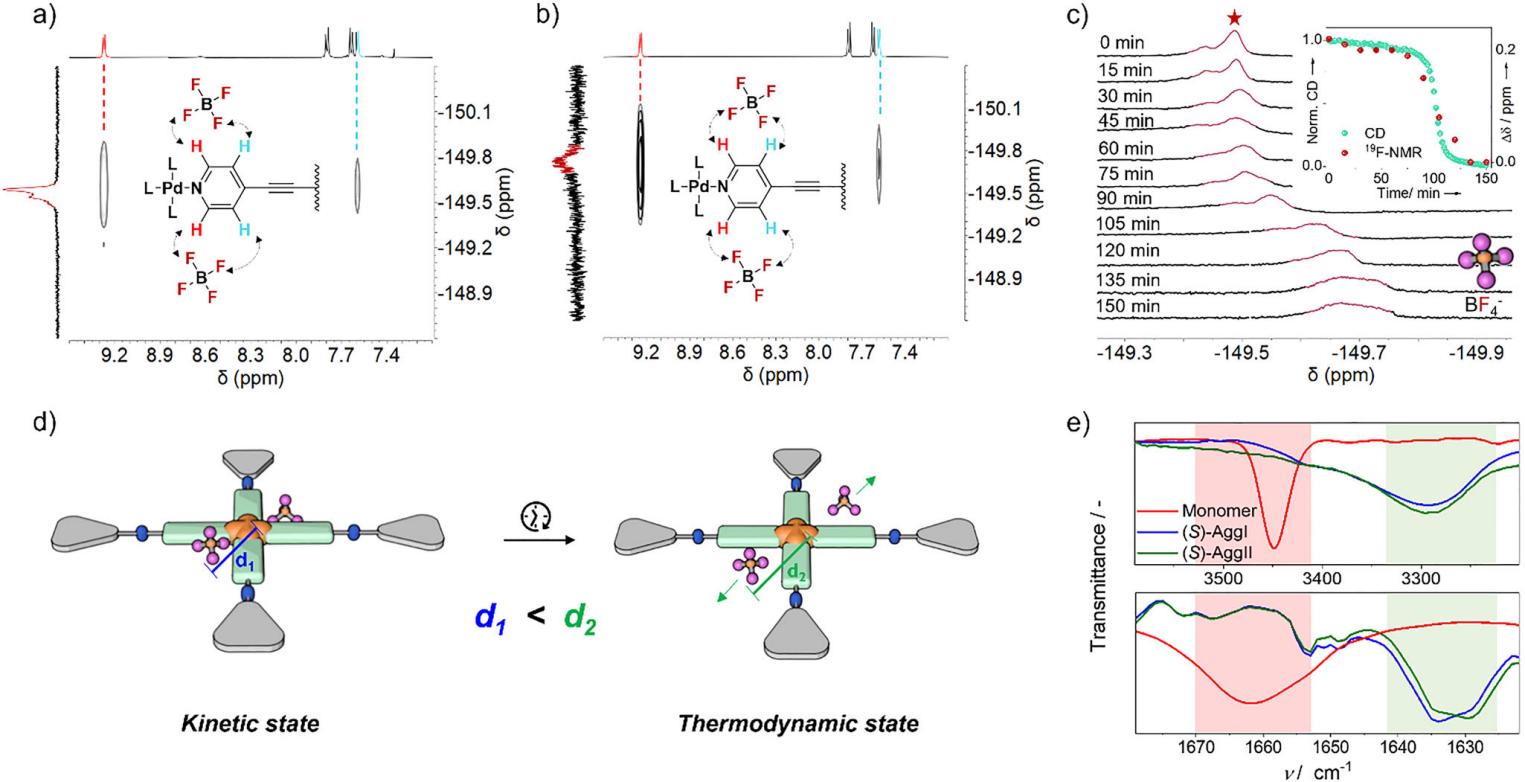
(a) Partial ^1^H–^19^F HOESY NMR (600 MHz) of freshly prepared **(*S*)‐AggI** in CD_2_Cl_2_:MCH‐d_14_ (1:1) (*c* = 4 mM, *T* = 278 K). (b) Partial ^1^H–^19^F HOESY NMR (600 MHz) of aged **(*S*)‐AggII** in CD_2_Cl_2_:MCH‐d_14_ (1:1) (*c* = 4 mM, *T* = 298 K). (c) Time‐dependent ^1^ ^9^F‐NMR in CD_2_Cl_2_:MCH‐d_14_ (1:1) (*c* = 0.5 mM, *T* = 278 K) and inset of kinetic profile in CD revealing the aggregate transformation over time (*λ* = 333 nm). (d) Schematic illustration of the proposed supramolecular arrangement, indicating the preferential localization of the counterion within the inner region of the ligand framework, as suggested by HOESY NMR experiments. (e) FTIR measurements in pure DCM (red) and fresh solution in MCH:DCM (8:2) as well as aged solution in MCH:DCM (8:2) at *c* = 0.5 mM and 298 K. N‐H region (top) and C = O region (bottom).

Since higher concentrations are required for multidimensional NMR experiments, solvent‐dependent CD measurements were performed under the same conditions to identify a suitable solvent composition for the characterization of the aggregated state. The CD spectrum remains silent in pure CD_2_Cl_2_ or upon addition of MCH‐d_14_ up to a 7:3 ratio, whereas a CD signal emerges at a 6:4 ratio and becomes fully developed at 1:1, confirming the formation of the kinetically controlled aggregate **(*S*)‐AggI** at higher concentrations (Figure S34d).

Direct evidence for the spatial proximity between the counterion and the molecular framework is provided by ^1^H–^1^ ^9^F HOESY experiments performed in CD_2_Cl_2_:MCH‐d_14_ (1:1) at 4 mM on freshly prepared samples at 278 K and on aged samples at room temperature (Figure 4a,b). Complementary CD measurements recorded under the same conditions confirm the presence of the two aggregated states at their respective temperatures (Figure S35a). For the freshly prepared solution at 278 K **((*S*)‐AggI)**, clear cross‐peaks are observed between the BF_4_^−^ resonance and the pyridine protons, confirming the proximity of the counterion to the aromatic core (Figure 4a). A strong correlation is detected for the α‐proton of the pyridine ring (δ = 9.28 ppm) with the BF_4_^−^ signal (δ ≈ −149.5 ppm), whereas a weaker cross‐peak is observed for the β‐proton (δ = 7.59 ppm), consistent with a greater spatial separation. Similar cross‐peaks between the BF_4_^−^ resonance and the pyridine protons are observed for **(*S*)‐AggII**, confirming that the counterion remains in close proximity to the aromatic core in both aggregated states. However, a subtle but significant difference is observed: in the freshly prepared sample, corresponding to the kinetically controlled aggregate **(*S*)‐AggI**, the cross‐peak involving the β‐proton is weaker, whereas in the aged sample corresponding to the thermodynamically favored aggregate **(*S*)‐AggII**, this correlation becomes slightly more pronounced. In addition, a different chemical shift of the ^1^ ^9^F resonance is observed for both aggregates (−149.5 ppm for **(*S*)‐AggI** versus −149.7 ppm for **(*S*)‐AggII**), indicating a change in the local environment of the counterion. These observations suggest subtle changes in counterion positioning between (***S*)‐AggI** and (***S*)‐AggII**, supporting the role of counterion dynamics in directing the competing assembly pathways.

To monitor the evolution of the counterion during the assembly process, time‐dependent ^1^ ^9^F NMR experiments were performed in CD_2_Cl_2_:MCH‐d_14_ (1:1) at a concentration of 0.5 mM and 278 K (Figure 4c). The BF_4_^−^ signal was recorded every 15 min. At the initial stage (0 min), corresponding to aggregate **(*S*)‐AggI**, the ^1^ ^9^F resonance appears at −149.5 ppm and remains nearly unchanged over the first 75 min. After ∼90 min, a noticeable shift is observed (−149.55 ppm), which evolves to −149.60 ppm at 120 min and finally stabilizes at −149.70 ppm after 135–150 min, consistent with the formation of **(*S*)‐AggII**. Time‐dependent CD measurements performed under identical conditions show that the supramolecular transformation occurs on the same timescale, with a clear change in the chiroptical signal after approximately 90 min (inset of Figure 4c). Plotting the temporal evolution of the ^1^ ^9^F NMR signal corresponding to the main resonance (highlighted with an asterisk in the inset of Figure 4c, initial δ = −149.5 ppm) reveals a kinetic profile that closely matches the CD evolution, confirming that both techniques monitor the same **(*S*)‐AggI→(*S*)‐AggII** transformation. These results demonstrate that the local environment and position of the BF_4_^−^ counterion change during the structural evolution of the supramolecular assembly. A schematic illustration is provided in Figure 4d, highlighting a more localized positioning of the counterion in **(*S*)‐AggI** and a slightly more displaced arrangement relative to the metal center in **(*S*)‐AggII**, consistent with the spectroscopic observations.

To further elucidate whether counterion positioning influences the hydrogen bonding interactions governing the formation of the two aggregated states, FTIR measurements were performed at 0.5 mM (Figure 4e). In DCM, monomeric **(*S*)‐1** displays an amide N–H stretching band at 3449 cm^−^^1^ and a C═O stretching band at 1662 cm^−^^1^, characteristic of free, non‐hydrogen‐bonded amide groups. In MCH:DCM (8:2), both bands shift to lower wavenumbers, indicating hydrogen‐bond formation. For **(*S*)‐AggI**, the N─H stretching appears at 3291 cm^−^^1^, whereas for **(*S*)‐AggII** it is slightly shifted to 3288 cm^−^^1^. A similar trend is observed for the amide carbonyl stretch, which shifts from 1634 cm^−^^1^ in **(*S*)‐AggI** to 1630 cm^−^^1^ in **(*S*)‐AggII**. The lower wavenumbers observed for **(*S*)‐AggII** indicate slightly stronger hydrogen‐bonding interactions in the thermodynamically favored aggregate.

To obtain further support for the proposed packing modes of the two supramolecular aggregates, semiempirical tight‐binding calculations (GFN2‐xTB) were performed [76]. Monomeric structures were first constructed and fully optimized in the absence of counteranions. Subsequently, based on previous reports on structurally related systems [66, 77] and on the through‐space correlations observed in the ^1^H–^1^ ^9^F HOESY experiments, the BF_4_^−^ anions were positioned near the *α*‐ and *β*‐pyridyl hydrogen atoms of the ligand. The resulting ion‐paired geometries were then reoptimized in dichloromethane (DCM) at the same level of theory.

Based on the optimized monomer geometry, a dimeric assembly was constructed using Avogadro 1.2.0. Distance constraints were applied to the BF_4_^−^ anions to prevent ion pairing with the Pd center in hexane, thereby affording **(*S*)‐AggI**, whereas **(*S*)‐AggII** was obtained by laterally displacing the counterions by approximately 1 Å relative to the Pd center followed by reoptimization under the same constraints. This displacement increases pyridine coplanarity and leads to the distinct packing motif observed for **(*S*)‐AggII**. Higher‐order oligomers, ranging from dimers to decamers, were subsequently constructed from the optimized geometries (Figures S36–S39). In all cases, the relative orientation of the monomeric units and the directionality of the hydrogen‐bonding interactions were preserved throughout the assemblies.

Structural comparison of the decamers of both assemblies (Tables S4–S7) reveals that **(*S*)‐AggI** adopts (*M*)‐helicity, arising from the transfer of chiral information from the (*S*)‐stereocenters in the side chains of **1** to the supramolecular stacks. In this structure, the BF_4_^−^ counterions remain in close proximity to the Pd center (B–Pd distance ≈ 4.77 Å) (Figure 5b), limiting pyridine coplanarity (*ϕ* = –35.6°) (Figure 5a). Additionally, the chiral side chains introduce a slight torsion between adjacent monomer units (*θ* = –14.2°), thereby defining the overall packing of the assembly. The reduced coplanarity and elongated hydrogen‐bond distances hinder the formation of extended supramolecular fibers, thereby accounting for the experimentally observed formation of nanoparticle‐like assemblies with lower cooperativity and reduced thermodynamic stability compared to **(*S*)‐AggII**. By contrast, **(*S*)‐AggII** is obtained when the BF_4_^−^ counterions are displaced from the Pd center (B–Pd distance ≈ 5.60 Å) (Figure 5d), allowing the pyridine ligands to adopt a more coplanar conformation (*ϕ* = –66.3°) (Figure 5c). This increased coplanarity reorganizes the relative orientation of the pyridine units and inverts the supramolecular helicity from (*M*)‐ to (*P*)‐oriented. In this packing mode, the helicity is no longer dictated solely by the (*S*)‐stereocenters of **1**, but is additionally modulated by the position of the counterions. The enhanced coplanarity shortens the hydrogen‐bonding distances, increases cooperativity, and stabilizes the assembly, consistent with the experimentally observed 1D morphologies. Finally, sTDA‐xTB calculations [78] performed on hexamers of **(*S*)‐AggI** and **(*S*)‐AggII** (Figures S40c and S41c) reproduce the main features of the experimental CD spectra, supporting the existence of two distinct packing modes. Stabilization energies computed for decamers at the GFN2‐xTB level (Figures S40b and S41b) further indicate a cooperative growth mechanism for both assemblies. Ultimately, the observed differences in asymmetry amplification (Figures S42–S46) further support that counterion‐dependent supramolecular packing governs not only helical sense but also the propagation of chiral information within supramolecular polymers.

**FIGURE 5:**
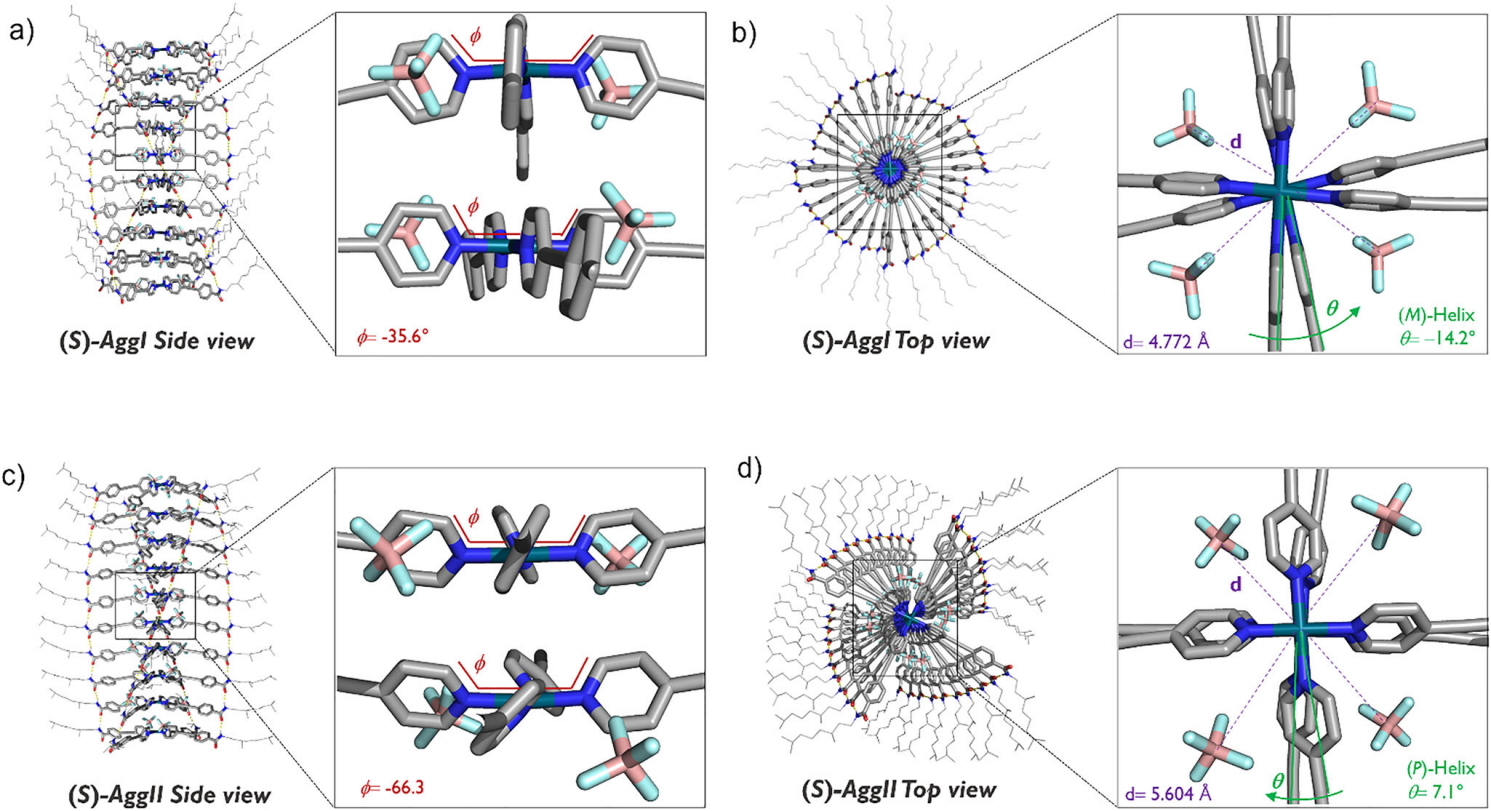
Optimized decamer structures of the two supramolecular aggregates computed at the GFN2‐xTB level. (a) Side view and (b) top view of **(*S*)‐AggI**. c) Side view and d) top view of **(*S*)‐AggII**. Insets in the side views highlight the pyridine coplanarization angle (ϕ), while the top views show the torsional angle (θ) between adjacent monomers and the distances between the BF_4_^−^ counteranions and the Pd center.

## 3 Conclusion

In summary, we have demonstrated that a single counterion can play an active role in pathway‐dependent self‐assembly and supramolecular helicity. To investigate these effects, we have designed (*S*)‐ and (*R*)‐chiral dicationic tetrapyridyl Pd(II) complexes bearing two BF_4_^−^ counterions (**(*S*)‐1** and **(*R*)‐1**), together with a reference achiral compound (**2**). The target complexes were strategically engineered with a cross‐shaped π‐extended aromatic surface, amide groups, and chiral side chains to promote self‐assemble into chiral supramolecular nanostructures in nonpolar media through a combination of aromatic and hydrogen bonding interactions. In MCH:DCM (8:2) mixtures, **(*S*)‐1** initially self‐assembles into a metastable aggregate (**AggI**) with nanoparticle‐like morphology, which undergoes structural reorganization over time into thermodynamically favored supramolecular polymers (**AggII**), accompanied by an inversion of the chiroptical signal. This transformation proceeds through competing pathways and is strongly dependent on the preparation protocol. Upon rapid solvent exchange from DCM into MCH (solvophobic quenching), further favored at low temperature, the initial counterion arrangement is retained during the early stages of self‐assembly, enabling the isolation of the metastable aggregate. Over time, however, the increasing packing constraints associated with π−π stacking and hydrogen bonding interactions favor changes in counterion positioning and molecular packing, leading to the thermodynamically favored aggregate. This subtle counterion relocation alters the coplanarity and packing of the pyridyl ligands and ultimately results in pathway‐dependent supramolecular chirality. These observations suggest that the interplay between charge desolvation, counterion dynamics, and supramolecular packing constitutes a key driving force underlying the observed pathway complexity. While the present work deliberately isolates the role of a single counterion, extending this approach to related coordination complexes bearing different weakly coordinating counterions will provide valuable insights into the generality of the proposed mechanism. In addition to elucidating the molecular origin of the observed helical inversion, this work demonstrates that counterions should not be regarded as passive charge‐balancing species, but as dynamic structural elements capable of directing supramolecular organization. Our findings establish counterion dynamics as a powerful design element for controlling pathway‐dependent supramolecular self‐assembly and supramolecular chirality, paving the way toward adaptive chiral materials based on coordination‐driven self‐assembly.

## Author Contributions

**Camila Montañez‐Moyano**: investigation, writing – original draft, methodology, formal analysis, validation, visualization. **Timo Krüger**: investigation, methodology, validation, visualization, writing – review and editing, formal analysis. **Alejandro Martínez‐Manjarrés**: writing – review and editing, visualization, methodology. **Gustavo Fernández**: writing – review and editing, funding acquisition, conceptualization, validation, project administration, supervision, resources.

## Conflicts of Interest

The authors declare no conflicts of interest.

## Supporting information




**Supporting File**: anie73859‐sup‐0001‐SuppMat.docx.

## Data Availability

The data that supports the findings of this study are available in the Supporting Information of this article.
