# Supplementary material for: Counterion Dynamics in Chiral Supramolecular Polymerization
Source: Angew Chem Int Ed Engl. 2026 Jul 31;65(40):e1807439. doi: 10.1002/anie.1807439 (PMC13618277; doi:10.1002/anie.1807439)
Supplement: Supplementary file 1 [file ANIE-65-e1807439-s001.docx]

Supporting Information
©Wiley-VCH 2021
69451 Weinheim, Germany

Counterion Dynamics in Chiral Supramolecular Polymerization

Camila Montañez-Moyano,^+^ Timo Krüger,^+^ Alejandro Martínez-Manjarrés and Gustavo Fernández*^[a]^

[a] Universität Münster, Organisch-Chemisches Institut, Corrensstraße 36, 48149 Münster (Germany).

E-mail: [fernandg@uni-muenster.de](mailto:fernandg@uni-muenster.de)

**Abstract:** Counterions have been studied in a broad range of chemical and biological systems, and their effects are typically evaluated by comparing different ionic species. However, the dynamic effects of a single counterion within a given system remain poorly understood. Herein, we demonstrate that counterion dynamics governs assembly pathways and chirality in supramolecular polymerization. To investigate this phenomenon, we designed dicationic tetrapyridyl Pd(II) complexes bearing two BF₄⁻ counterions, amide groups for hydrogen bonding, and either *S*- or *R*-chiral side chains, and studied their supramolecular polymerization in nonpolar media. Under kinetic conditions, the *S*-chiral complex initially self-assembles into nanoparticle-like metastable aggregates (**AggI**), which over time evolve into thermodynamically controlled supramolecular polymers (**AggII**) with inverted chiroptical signal through a competitive pathway. Spectroscopic, microscopic, and computational studies indicate that subtle changes in the position of the BF₄⁻ counterions modulate the packing of the π-extended pyridine ligands and the hydrogen-bonding arrangement, giving rise to distinct asymmetry amplification behaviour and supramolecular chirality. These findings demonstrate that a single counterion can direct pathway-dependent supramolecular polymerization into distinct helical states, highlighting the crucial role of charge desolvation and dynamics in governing supramolecular self-assembly.

Supporting Information

**Table of Contents**

[**Experimental section**	2](#_Toc231997374)

[Materials and methods	2](#_Toc231997375)

[Synthesis and Characterization	3](#_Toc231997376)

[**Supplementary Experiments/Figures**	14](#_Toc231997377)

[Self-assembly Studies	14](#_Toc231997378)

[Denaturation Model for Supramolecular Polymerization	17](#_Toc231997379)

[Thermodynamic and kinetic Characterization	17](#_Toc231997380)

[Morphological studies and analysis of packing	20](#_Toc231997381)

[Theoretical calculations	22](#_Toc231997382)

[**References**	34](#_Toc231997383)

## **Experimental section**

## Materials and methods

**General.** All solvents were dried according to standard procedures. Reagents were used as purchased. All air-sensitive reactions were carried out under argon atmosphere. For all spectroscopic and photophysical measurements, spectroscopic grade solvents were used.

**Nuclear magnetic Resonance (NMR) Spectroscopy.** ^1^H-NMR and ^13^C-NMR spectra were recorded on a Bruker Avance 400 (^1^H: 500 MHz, ^13^C: 126 MHz) at a standard temperature of 298 K using deuterated solvents. The recorded spectra were referenced to the remaining resonance signals of the deuterated solvents. The coupling constant *J* of the measured spin multiplets is given in Hertz (Hz) and the chemical shifts (*δ*) are given in ppm relative to trimethylsilane (^1^H, 0.0 ppm). Multiplicities for proton signals are abbreviated as s (singlet), d (doublet), t (triplet), q (quadruplet) and m (multiplet).

**Mass Spectrometry.** Electrospray ionization (ESI) mass spectra were recorded on a Bruker MicroToF instrument. The signals are described by their mass/charge ratio (m/z) in u.

**UV-vis Spectroscopy**. UV-vis absorption spectra were recorded on a JASCO V-770/750 with a spectral bandwidth of 1.0 nm and a scan rate of 1000 nm min^-1^. All experiments were carried out using quartz cuvettes with an optical path of 1 cm. All measurements were performed in commercially available solvents of spectroscopic grade.

**Fluorescence Spectroscopy.** All emission spectra were acquired on a JASCO FP-8500 with a spectral bandwidth of 1.0 mm and a scan rate of 500-1000 min^-1^. Quartz cuvettes with an optical length of 1 cm were used. All measurements were performed in commercially available solvents of spectroscopic grade.

**CD spectroscopy.** CD spectra were performed on a JASCO J-1500 spectropolarimeter equipped with a Peltier module as a temperature controller. The data was recorded with a spectral bandwidth of 1.0 nm and a scanning speed of 500 nm min^-1^. All experiments were carried out using quartz cuvettes with an optical path of 1 cm. All measurements were performed in commercially available solvents of spectroscopic grade.

**Atomic force microscopy (AFM)** images have been recorded on a Bruker AXS Multimode 8 SPM System in soft tapping mode. The used cantilevers were Bruker NCHV-A with an average spring constant of 42 N·m^-1^, an average resonance frequency of 320 kHz, an average length of 117 µm, an average width of 33 µm and an average tip radius of 8 nm. Samples have been prepared by depositing 10 µM solutions of **(*S*)-AggI** or **(*S*)-AggII** in MCH/DCM 8:2 (*v*/*v*) on mica surface, followed by a waiting time of 15 or 20 s for **(*S*)-AggI** and **(*S*)-AggII** respectively, spin-coating for 30 s at 1000 rpm, and drying of the surface overnight before starting the respective measurement.

**Nano-Differential scanning calorimetry (Nano DSC).** Nano-Differential scanning calorimetry measurements in solution were performed on a TA instruments nano-DSC at a pressure of 3 atm in a 600 µL platinum capillary cell, for sample and reference, using a heating-cooling rate of 1 ºC min^‒1^.

**Scanning Electron Microscopy (SEM).** Microscopy samples were prepared by drop-casting a solution of the respective aggregates onto silicon wafers, followed by solvent evaporation at 298 K. SEM images were recorded using a Thermo Fisher Scientific Phenom ProX Desktop SEM. Images were acquired with an acceleration voltage of 5–15 kV, using either a backscattered-electron detector (BSD) or a secondary-electron detector (SED). All samples were deposited onto Si wafer surfaces and allowed to dry under ambient conditions.

**Dynamic and Static Light Scattering**. DLS and SLS measurements were carried out using a CGS-3 Compact Goniometer System (ALV) equipped with LSE-5004 Light Scattering Electronics (22 mW HeNe laser, 633 nm) and a Multiple Tau Digital Correlator unit (ALV). Solvents were filtered prior to sample preparation through 0.45 µm nylon or Teflon filters. SLS data were analyzed using the ALV-Fit & Plot software.

## Synthesis and Characterization

Compounds **3** to **6** were prepared following previously reported procedures and exhibited similar spectroscopic features.^[1–3]^

**Scheme S1.** Synthesis route to obtain ligands **(*R*)-L_1_, (*S*)-L_1,_** **L_2_** and complexes **(*S*)-1, (*R*)-1, 2**.

**(*R*)-N-(3,7-dimethyloctyl)-4-(pyridin-4-ylethynyl)benzamide ((*R*)-L_1_).**

4-(pyridin-4-ylethynyl)benzoic acid (190.0 mg, 0.85 mmol, 1 eq.) was dissolved in DCM and EDC∙HCl (325.89 mg, 1.70 mmol, 2 eq.) together with DMAP (125.0 mg, 1.02 mmol, 1.2 eq.) were added. The mixture was stirred for 10 min at room temperature, after which a few drops of DMF were added and stirring was continued for additional 5 min. Afterwards, (*R*)-3,7-dimethyloctan-1-amine (200.0 mg, 1.27 mmol, 1.5 eq.) was added and the reaction mixture was stirred for 24 h at RT. After the addition of water, the phases were separated, and the aqueous layer was extracted with DCM three times. The combined organic layers were washed with brine and dried over anhydrous MgSO₄. The solvent was removed under reduced pressure, and the crude mixture was purified by column chromatography using DCM → DCM/MeOH (99:1, v/v), followed by a second column with *n*-pentane/EtOAc (6:4, v/v). The product was obtained as brownish solid (95.8 mg, 0.26 mmol, 31%).

**^1^H NMR** (500 MHz, CD_2_Cl_2_) *δ* (ppm) = 8.60 (d, 2H), 7.75 (d, J = 0.6 Hz, 2H), 7.63 (d, J = 0.5 Hz, 2H), 7.41 (d, 2H), 6.14 (s, 1H), 3.45 (m, 2H), 1.68 – 1.60 (m, 1H), 1.54 (m, J = 6.6 Hz, 2H), 1.42 (m, J = 13.7, 8.2, 6.0 Hz, 1H), 1.35 (d, J = 4.1 Hz, 3H), 1.17 (m, 3H), 0.95 (d, J = 6.6 Hz, 3H), 0.88 (d, 6H).

**^13^C NMR** (126 MHz, CD_2_Cl_2_) *δ* (ppm) = 166.08, 149.88, 135.39, 131.92, 130.73, 126.94, 125.41, 92.56, 88.41, 39.22, 38.21, 37.11, 36.68, 30.76, 27.96, 24.63, 22.36 (d, J = 12.2 Hz), 19.27.

**HR-MS** (ESI, CH_3_OH/CHCl_3_): m/z 385.2250 [M+Na] ^+^, calculated for 385.2254 C_24_H_30_N_2_ONa.

**Figure S1.**^1^H NMR (500 MHz, CD_2_Cl_2_) spectrum of **(*R*)-L_1_**.

 

**Figure S2.**^13^C NMR (126 MHz, CD_2_Cl_2_) spectrum of **(*R*)-L_1_**.

**(*S*)-N-(3,7-dimethyloctyl)-4-(pyridin-4-ylethynyl)benzamide ((*S*)-L_1_).**

4-(pyridin-4-ylethynyl)benzoic acid (190.0 mg, 0.85 mmol, 1 eq.) was dissolved in DCM and EDC∙HCl (325.89 mg, 1.70 mmol, 2 eq.) together with DMAP (125.0 mg, 1.02 mmol, 1.2 eq.) were added. The mixture was stirred for 10 min at RT, then few drops of DMF were added and stirred additionally for 5 min. Afterwards (*S*)-3,7-dimethyloctan-1-amine (200.0 mg, 1.27 mmol, 1.5 eq.) was added and the reaction mixture was stirred for 24 h at RT. After the addition of water, the phases were separated, and the aqueous layer was extracted with DCM three times. The combined organic layers were washed with brine and dried over anhydrous MgSO₄. The solvent was removed under reduced pressure, and the crude mixture was purified by column chromatography using DCM → DCM/MeOH (99:1, v/v), followed by a second column with n-pentane/EtOAc (6:4, v/v). The product was obtained as brownish solid (95.8 mg, 0.26 mmol, 31%).

**^1^H NMR** (500 MHz, CD_2_Cl_2_) *δ* (ppm) = 8.59 (d, 2H), 7.76 (d, J = 0.6 Hz, 2H), 7.64 (d, J = 0.6 Hz, 2H), 7.41 (d, J = 1.6 Hz, 2H), 6.18 (s, 1H), 3.47 (m, 2H), 1.62 (d, J = 2.0 Hz, 2H), 1.54-1.50 (m, 2H), 1.44 (m, 1H), 1.31-1.25 (m, J = 1.1 Hz, 2H), 1.18 (d, 3H), 0.96 (d, 3H), 0.88 (d, 6H).

**^13^C NMR** (126 MHz, CD_2_Cl_2_) *δ* (ppm) = 166.31, 150.09, 135.62, 132.13, 130.96, 127.16, 125.63, 125.15, 92.79, 88.62, 39.43, 38.43, 37.81, 37.33, 37.32, 36.95, 36.90, 30.97, 30.86, 28.18, 24.85, 24.83, 22.63, 22.53, 19.49, 19.42.

**HR-MS** (ESI, CH_3_OH/CHCl_3_): m/z 385.2250 [M+Na] ^+^, calculated for 385.2254 C_24_H_30_N_2_ONa.

**Figure S3.**^1^H NMR (500 MHz, CD_2_Cl_2_) spectrum of **(*S*)-L_1_**.

**Figure S4.**^13^C NMR (126 MHz, CD_2_Cl_2_) spectrum of **(*S*)-L_1_**.

**N-decyl-4-(pyridin-4-ylethynyl)benzamide (L_2_).**

4-(pyridin-4-ylethynyl)benzoic acid 5 (200.0 mg, 0.9 mmol, 1 eq.) was dissolved in DCM and EDC∙HCl (345.0 mg, 1.8 mmol, 2 eq.) together with DMAP (22.0 mg, 0.18 mmol, 0.2 eq.) were added. The mixture was stirred for 10 min at 0 °C, after which 1-aminodecane (283.0 mg, 1.8 mmol, 2 equiv) was added, and the reaction mixture was stirred for 24 h at room temperature. Following the addition of water, the phases were separated, and the aqueous layer was extracted with DCM three times. The combined organic layers were washed with brine and dried over anhydrous MgSO₄. The solvent was removed under reduced pressure, and the crude mixture was purified by column chromatography using DCM → DCM/MeOH (99:1, v/v). The product was obtained as a beige solid (146.0 mg, 0.4 mmol, 45%).

**^1^H-NMR** (400 MHz, CD_2_Cl_2_) *δ* (ppm) = 8.60 (d, ^3^J_HH_ = 6.1 Hz, 2H), 7.76 (d, ^3^J_HH_ = 8.4 Hz, 2Hd), 7.63 (d, ^3^J_HH_ = 8.3 Hz, 2H), 7.40 (d, ^3^J_HH_ = 5.2 Hz, 2H), 6.22 (s, NH, 1H), 3.41 (td, ^3^J_HH_ = 7.3 Hz, ^3^J_HH_ = 5.8 Hz 2H). 1.67 – 1.54 (m, 2H), 1.40 – 1.32 (m, 4H) 1.27 (s, 10H), 0.92 – 0.83 (m, 3H).

**^13^C NMR** (126 MHz, CD_2_Cl_2_) *δ* (ppm) = 166.08, 149.88, 135.39, 131.92, 130.73, 126.94, 125.41, 92.56, 88.41, 39.22, 38.21, 37.11, 36.68, 30.76, 27.96, 24.63.

**HR-MS** (ESI, CH_3_OH/CHCl_3_): m/z 385.2253 [M+Na] ^+^, calculated for 385.2255 C_24_H_30_N_2_ONa.

**Figure S5.**^1^H NMR (400 MHz, CD_2_Cl_2_) spectrum of **L_2_**.

**Figure S6.**^13^C NMR (126 MHz, CD_2_Cl_2_) spectrum of **L_2_**.

**(*R*)-N-(3,7-dimethyloctyl)-4-(pyridin-4-ylethynyl)benzamide palladium tetrafluoroborate ((*R*)-1).**

(*R*)-N-(3,7-dimethyloctyl)-4-(pyridin-4-ylethynyl)benzamide **((*R*)-L_1_)** (50.0 mg, 0.14 mmol, 4 eq.) together with [Pd(ACN)_4_(BF_4_)_2_] (15.32 mg, 0.03 mmol, 1 eq.) were dissolved in dry DCM. The mixture was stirred at RT for 5 h. Et_2_O was then added as an overlayer and the mixture placed in the fridge for precipitation overnight. After the precipitate was filtered off, the solid was redissolved in DCM and again precipitated for further purification. The complex was obtained as a beige solid (38.7 mg, 0.022 mmol, 65%).

**^1^H-NMR** (600 MHz, CD_2_Cl_2_) *δ* (ppm) = 9.21 (d, ^3^*J*_HH_ = 6.8 Hz, 8H), 7.75 (d, ^3^*J*_HH_ = 8.8 Hz, 8H), 7.61 (d, ^3^*J*_HH_ = 8.7 Hz, 8H), 7.56 (d, ^3^*J*_HH_ = 6.0 Hz, 8H), 6.12 (d, ^3^*J*_HH_ = 8.3 Hz, 4H), 3.50 – 3.37 (m, 8H), 1.66 – 1. 63 (m, 4H), 1.57 – 1.47 (m, 8H), 1.42 – 1.4 (m, 4H),1.35 – 1.21 (m, 16H) 1.20 – 1.10 (m, 12H), 0.93 (d, ^3^*J*_HH_ = 7.4 Hz, 10H) 0.86 (d, ^3^*J*_HH_ = 7.4 Hz, 22H).

**^13^C-NMR** (151 MHz, CD_2_Cl_2_) *δ* (ppm) = 166.28, 151.58, 136.79, 136.30, 132.79, 129.25, 127.46, 123.98, 99.01, 86.69, 39.63, 38.68, 37.52, 37.07, 31.17, 28.38, 25.05, 22.83, 22.73, 19.69.

**^19^F NMR** (470 MHz, CD_2_Cl_2_) *δ* (ppm) = -149.62 (d, J = 1.7 Hz), -149.66 – -149.69 (m).

**HR-MS (ESI, CHCl_3_):** m/z 1728.8719 [M+H] ^+^, 1728.8526 calculated for PdC_96_H_120_N_8_O_4_B_2_F_8_

**Figure S7.**^1^H NMR (600 MHz, CD_2_Cl_2_) spectrum of **(*R*)-1**.

**Figure S8.**^13^C NMR (151 MHz, CD_2_Cl_2_) spectrum of complex **(*R*)-1**.

**Figure S9.**^19^F NMR (470 MHz, CD_2_Cl_2_) spectrum of complex **(*R*)-1**.

**(*S*)-N-(3,7-dimethyloctyl)-4-(pyridin-4-ylethynyl)benzamide palladium tetrafluoroborate ((*S*)-1).**

(*S*)-N-(3,7-dimethyloctyl)-4-(pyridin-4-ylethynyl)benzamide **((*S*)-L_1_)** (103,0 mg, 0.28 mmol, 4 eq.) together with [Pd(ACN)_4_(BF_4_)_2_] (31,6 mg, 0.07 mmol, 1 eq.) were dissolved in dry DCM. The mixture was stirred at RT for 5 h. Et_2_O was then added as an overlayer and the mixture placed in the fridge for precipitation overnight. After the precipitate was filtered off, the solid was redissolved in DCM and again precipitated for further purification. The complex was obtained as a beige solid (81.31 mg, 0.047 mmol, 67%).

**^1^H NMR** (600 MHz, CD_2_Cl_2_) *δ* (ppm) = 9.22 (d, 8H), 7.74 (d, 8H), 7.62 (d, 8H), 7.56 (d, J = 6.8 Hz, 8H), 6.10 (s, 4H), 3.43 (m, 8H), 1.62 (s, 2H), 1.54 – 1.48 (m, 16H), 1.43 (m, 5H), 1.33 (m, 15H), 1.16 (d, J = 6.9 Hz, 10H), 0.94 (d, 10H), 0.85 (d, 18H).

**^13^C NMR** (151 MHz, CD_2_Cl_2_) *δ* (ppm) = 165.83, 151.13, 136.34, 135.85, 132.34, 128.80, 127.01, 123.53, 98.56, 86.24, 39.18, 38.23, 37.07, 36.62, 30.72, 27.93, 24.60, 22.38, 22.28, 19.24.

**^19^F NMR** (470 MHz, CD_2_Cl_2_) *δ* (ppm) = -149.67 (d, J = 25.1 Hz).

**HR-MS (ESI, CHCl_3_):** m/z 1728.8720 [M+H] ^+^, 1728.8526 calculated for PdC_96_H_120_N_8_O_4_B_2_F_8_

**Figure S10.**^1^H NMR (600 MHz, CD_2_Cl_2_) spectrum of **(*S*)-1**.

**Figure S11.**^13^C NMR (151 MHz, CD_2_Cl_2_) spectrum of complex **(*S*)-1**.

**Figure S12.**^19^F NMR (470 MHz, CD_2_Cl_2_) spectrum of complex **(*S*)-1**.

**Tetra-N-decyl-4-(pyridin-4-ylethynyl)benzamide palladium tetrafluorobonate (2).**

N-decyl-4-(pyridin-4-ylethynyl)benzamide **(L_2_)** (100.0 mg, 0.28 mmol, 4 eq.) together with [Pd(ACN)_4_(BF_4_)_2_] (30.6 mg, 0.07 mmol, 1 eq.) were dissolved in dry DCM. The mixture was stirred at RT for 5 h. Et_2_O was then added as an overlayer and the mixture placed in the fridge for precipitation overnight. After the precipitate was filtered off, the solid was redissolved in DCM and again precipitated for further purification. The complex was obtained as a beige solid (70.0 mg, 0.04 mmol, 59%).

**^1^H NMR** (500 MHz, CD_2_Cl_2_) *δ* (ppm) = 9.22 (d, J = 7.0 Hz, 8H), 7.75 (d, J = 8.6 Hz, 8H), 7.62 (d, 8H), 7.56 (d, J = 7.0 Hz, 8H), 6.17 (s, 4H), 3.43 – 3.37 (m, 8H), 1.61-1.58 (d, J = 7.2 Hz, 19H), 1.27 (m, 15H) 1.34 (m, 30H), 0.87 (d, 12H).

**^13^C NMR** (126 MHz, CD_2_Cl_2_) *δ* (ppm) = 165.88, 151.15, 136.36, 132.34, 128.81, 127.03, 123.54, 98.57, 40.12, 31.86, 29.57, 29.52, 29.52, 29.29, 29.27, 26.95, 22.64, 13.83.

**^19^F NMR** (470 MHz, CD_2_Cl_2_) *δ* (ppm) = -149.63, -149.68.

**HR-MS (ESI, CHCl_3_):** m/z 1728.8719 [M+H] ^+^, 1728.8526 calculated for PdC_96_H_120_N_8_O_4_B_2_F_8_

**Figure S13.**^1^H NMR (500 MHz, CD_2_Cl_2_) spectrum of **2**.

**Figure S14.**^13^C NMR (126 MHz, CD_2_Cl_2_) spectrum of complex **2**.

**Figure S15.**^19^F NMR (470 MHz, CD_2_Cl_2_) spectrum of complex **2**.

## **Supplementary Experiments/Figures**

## Self-assembly Studies

**Figure S16.** Sample preparation to obtain the kinetically controlled aggregate.

For the sample preparation, MCH: DCM mixtures were first prepared in the cuvette, considering the amount of stock solution (in DCM), and cooled to 273 K in a cooling bath. Aliquots of the stock solution were then added. To keep the effectiveness of the solvent quenching as constant as possible, 10 µL of stock solution were added each time depending on the desired concentration. The cuvette was kept in the cooling bath for 30 s of equilibration time, while suppressing transformation. Afterwards, the sample was set into the pre-set device, and the measurements were started immediately.

**Figure S17.** Solvent-dependent studies of **2** (*c* = 20 µM, 298 K). a) UV-vis absorption spectra and b) Emission spectra (*λ*_exc_. = 314 nm).

**Figure S18.** a,c) CD and b,d) UV-vis measurements of **(*S*)-1** and **(*R*)-1** (*c* = 20 µM, 298 K) at different MCH:DCM ratios.

**Figure S19.** VT-absorption studies of **(*S*)-1** in MCH:DCM 8:2 using a heating-cooling rate of 1 K min^-1^ (*c* = 20 µM). (a) Heating and (b) cooling experiments.

**Figure S20.** VT-spectroscopic studies of **(*R*)-1** in MCH:DCM 8:2 using a heating-cooling rate of 1 K min^-1^ (*c* = 20 µM). Heating: UV-vis (a), CD (b) and LD (c); subsequent cooling experiments: UV-vis (d), CD (e) and LD (f).

**Figure S21.** Temperature-dependent UV-vis measurements of **2** (*c* = 20 µM) in MCH:DCM 8:2 (*v*/*v*). a) heating, b) cooling) at 1 K/min^-1^. VT-CD measurements of **(*S*)-1** (*c* = 20 µM) in dry MCH:DCM 8:2 (*v*/*v*). a) heating, b) cooling) at 1 K/min^-1^.

**Figure S22:** Concentration-dependent CD measurements for complex **(*S*)-1** at 278 K prepared by solvophobic quenching at concentrations between *c* = 10 µM and 100 µM in MCH:DCM 8:2. a) **(*S*)-Agg-I** and b) **(*S*)-Agg-II**.

## Denaturation Model for Supramolecular Polymerization

The denaturation model^[4]^ is based on the concentration-dependent supramolecular polymerization equilibrium model by Goldstein, where the polymerization is described as a sequence of monomer addition equilibria.

[𝑃_𝑛_] = 𝐾_𝑛_ [𝑃_𝑛_ ‒ 1] [𝑋]	*(1)*

[𝑃𝑛 + 1] = 𝐾𝑒 [𝑃𝑛] [𝑋]	*(2)*

[𝑃𝑖] = 𝐾𝑒 [𝑃𝑖 ‒ 1] [𝑋]	*(3)*

For the cooperative model *K*_n_ < *K*_e_ while for an isodesmic process *K_n_* = *K*_e_. The concentration for each species *P*_i_ is given by [𝑃*𝑖*] = 𝐾 ^𝑖^ ^‒^ ^1^𝑛 [𝑋]^𝑖^ for 𝑖 ≤ 𝑛 and [𝑃_𝑖_] = 𝐾 ^𝑖 ‒ 𝑛^_𝑒_ 𝐾 ^𝑛 ‒ 1^_𝑛_ [𝑋]^𝑖^ for 𝑖 > 𝑛.

The dimensionless mass balance is obtained by inserting the dimensionless concentration 𝑝_𝑖_ = 𝑘_𝑒_[𝑃_𝑖_], the monomer concentration x= K_e_ [X] and the concentration of each species P_i_ (for i ≤ n): 𝑝_𝑖_ = 𝜎^𝑖−1^𝑥^𝑖^ and for 𝑖 > n: 𝑝_𝑖_ = 𝜎^𝑛−1^ 𝑥^𝑖^):

𝑥_𝑡𝑂𝑡_ = 𝜎^−1^ $\sum_{i=n}^{n} (i$𝜎𝑥) ^𝑖^ + 𝜎^𝑛−1^$\sum_{i=n+1}^{\infty} ix$^𝑖^	*(4)*

Both sums are evaluated by using standard expressions for converging series:

𝑥_𝑡𝑜𝑡_ = ($\frac{(\sigma{x)}^{n+1}(n\sigma x-n-1)}{{(\sigma x-1)}^{2}}+\frac{\sigma x}{{(\sigma x-1)}^{2}})-\sigma^{n-1}(\frac{x^{n+1}(nϰ-n-1}{{[x-1]}^{2}}$)	*(5)*

With 𝑥_𝑡𝑜𝑡_ = 𝐶_𝑡𝑜𝑡_𝑘_𝑒_ and *C*_𝑡𝑜𝑡_ = total monomer concentration

The sum solved by standard numerical methods (Matlabfzerosolver) yields the dimensionless monomer concentration 𝑥. The degree of 𝑖 > 1 aggregation results in:

𝛼_𝑎𝑔𝑔_ = $\frac{x_{tot}-x}{x_{tot}}$	*(6)*

Via 𝑘_𝑒_ = 𝑒(−$\frac{\Delta G^{0}}{\mathrm{RT}}$) the denaturation curves can be obtained with *f* defined as volume fraction of good solvent:

𝛥𝐺^0^ = 𝛥𝐺^0^ + 𝑚𝑓	*(7)*

It is assumed that the cooperativity factor 𝜎 is independent of the volume fraction and the *m* value for the elongation regime equals the *m* value for nucleation. The denaturation data needs to be transformed into the normalized degree of aggregation, if fitted to the supramolecular polymerization equilibrium model:

(𝑓) =$\frac{A\left( f \right)-A(f=0)}{A\left( f=1 \right)-A(f=0)}$	*(8)*

The optimization of the four needed parameters (*∆G_0_*, *m*, *σ* and *p*) to fit the experimental data (normalized degree vs. *f*) is done by the non-linear least-squares analysis using Matlab (lsqnonlinsolver). The data is then fitted with the non-linear least squared regression (Levenberg Marquardt algorithm).

The thermodynamic parameters for **(*S*)-AggI**, and **(*S*)-AggII** were obtained by fitting the respective experimental data to the nucleation- elongation model.

## Thermodynamic and kinetic Characterization

**Table S1.** Thermodynamic parameters associated with the supramolecular polymerization

|  | Δ*G*_298_ / kJ/mol^-1^ | *σ* | *m* values / kJ/mol |
| --- | --- | --- | --- |
| (*S*)-AggI | -49.6 | 1.8 x10^-2^ | 55.95 |
| (*S*)-AggII | -53.1 | 1.4 x10^-2^ | 58.78 |

**Figure S23**. Denaturation CD studies of **(*S*)-AggI** (DCM good and MCH poor solvent) at variable concentrations: a) 20 µM, b) 40 µM and c) 60 µM at 298 K.

**Figure S24.** Denaturation CD studies of **(*S*)-AggII** (DCM good and MCH poor solvent) at variable concentrations: a) 40 µM, b) 60 µM and c) 100 µM at 298 K.

**Figure S25.** Time-dependent CD studies of **(*S*)-1** at 30 μM in MCH:DCM 8:2 with varying temperatures: a) 293 K, b) 288 K and c) 283 K.

**Figure S26.** Time-dependent CD studies of **(*S*)-1** at 288 K in MCH:DCM 8:2 with varying concentrations: a) 100 μM, b) 250 μM and c) 500 μM.

**Figure S27.** Time-dependent CD measurements of **(*R*)-1** (*c* = 20 µM) in MCH:DCM 8:2 at (a) 278 K, (b) 288 K and (c) 298 K.

**Figure S28.** Fitting of the time-dependent CD measurements of **(S)-1** at *c* = 30 µM at different temperatures (283 K to 298 K) using AmyloFit ^[5]^. b) linear Eyring plot *ln*(*k*/*T*) versus 1/*T*, derived from the kinetic parameters obtained with AmyloFit.

Figure S28a shows the fitted curves of the experimental data obtained at different temperatures for a fixed concentration of *c* = 30 µM. From these fits, the rate constants were extracted using the AmyloFit script,^[5]^ revealing a clear temperature dependence. The corresponding values at each temperature are summarized in Table S2.

**Table S2.** Rate constants obtained from the mathematical fit of the time-dependent data.

| *T* / K | *k* / conc−^nc^ time^−2^ |
| --- | --- |
| 298 | 9.96∙10^12^ |
| 293 | 5.06∙10^12^ |
| 288 | 7.02∙10^11^ |
| 283 | 1.38∙10^11^ |

Using the data obtained at different temperatures, the linearized form of the Eyring equation (Eq. 9) was employed to determine the themodynamic parameters.

$$\ln\left( \frac{k}{T} \right)= \frac{-\Delta H^{\ddagger}}{R T}+\ln\left( \frac{k_{b}}{h} \right)+\frac{\Delta S^{\ddagger}}{R}$$

*(9)*

Accordingly, *ln*(*k/T*) was plotted as a function of 1/*T*, and a linear regression was applied. The resulting plot and fitted equation are shown in Figure S28b. From these data, the activation enthalpy, entropy, and Gibbs free energy were calculated; the corresponding values, together with their standard deviations, are summarized in Table S3.

**Table S3.** Thermodynamic Parameters calculated by the Eyring Equation (*9*), with the values obtained by the linear regression of the fits from the time-dependent CD measurements at *c* = 30 µM, from 288 K to 293 K

| Thermodynamic parameters | Calculated_Total_ | Calculated_Std_ |
| --- | --- | --- |
| ΔΔ*H*^‡^ | 9.30 kJ/ mol | ±1.21 kJ/ mol |
| ΔΔ*S*^‡^ | -0,14 kJ/mol∙K | ± 0.19 kJ/mol∙K |
| ΔΔ*G*°^‡^ | 50,93 kJ/ mol | ± 58.830 kJ/ mol |

## Morphological studies and analysis of packing

**Figure S29.** AFM height images of **(*S*)-AggI** prepared by depositing 10 µL (*c* = 20 µM in MCH:DCM 8:2) on a mica surface (spin-coated)

**Figure S30**. AFM height images of **(*S*)-AggII** prepared by depositing 10 µL (*c* = 20 µM in MCH:DCM 8:2) on a mica surface (spin-coated).

**Figure S31.** SEM images of **(*S*)-AggI** prepared by drop-casting 10 µL (*c* = 20 µM in MCH:DCM 8:2) on a silicon wafer.

**Figure S32**. SEM images of **(*S*)-AggII** prepared by drop-casting 10 µL (*c* = 20 µM in MCH:DCM 8:2) on a silicon wafer.

**Figure S33**. Angular-dependent DLS studies (*c* = 50 µM, 283 K, MCH:DCM (8:2, *v*/*v*)). Correlation function and corresponding size distribution for **(*S*)-AggI** (a,b) and **(*S*)-AggII** (c,d).

**Figure S34.** a) Molecular Structure of **(*S*)-1** with assignment of the relevant protons. b) Solvent-dependent ^1^H-NMR and c) ^19^F-NMR spectra at *c* = 0.5 mM and 298 K in various DCM:MCH mixtures (10% increments) ranging from 100 Vol-% DCM to 40 Vol-% DCM, showing the evolution of the chemical shifts of the indicated protons and fluorine nuclei, respectively. d) CD Spectra of **(*S*)-1** measured of the solutions used for NMR studies in (b).

Upon increasing the MCH fraction, the aromatic pyridyl protons (Ha and Hb) exhibit a gradual downfield shift rather than the shielding typically associated with π– π stacking. We attribute this behaviour to the proximity of the BF₄⁻ counterions to the pyridyl coordination environment, as supported by the HOESY experiments. The interaction between the fluorine atoms of the counterion and the pyridyl framework modifies the local electronic environment, leading to electron-density withdrawal around the α- and β-pyridyl protons and consequently to their deshielding. In contrast, the downfield shifts observed for the remaining aromatic protons are mainly associated with the increased polarization of the amide carbonyl groups upon hydrogen bond formation during aggregation. Similar deshielding effects have been reported for related metal-coordination systems in which the electronic influence of coordinated ligands and nearby counterions dominates over the shielding expected from π-stacking. ^[6,7]^

**Figure S35.** a) CD Spectra of **(*S*)-AggI** and **(*S*)-AggII** measured of the solutions used for HOESY NMR studies b) Partial ^1^H–^1^H ROESY-NMR spectra of (***S*)-1** in DCM: MCH 50:50 at 4 mM.

## Theoretical calculations

**Construction of Monomers and Oligomers**

All monomeric structures of **1** were constructed using Avogadro 1.2.0,^[8]^ and optimized geometries were obtained using Grimme’s GFN2-xTB method.^[9.10]^ Geometry optimizations were first performed in the absence of counteranions using dichloromethane (DCM) as solvent and applying default convergence criteria. BF₄⁻ counterions were subsequently positioned near the *α*- and *β*-pyridyl hydrogen atoms based on the through-space correlations observed in the NOESY experiments and on precedents reported for structurally related systems.^[11,12]^ The resulting ion-paired geometries were then reoptimized at the same level of theory in DCM.

Based on the optimized monomer geometry, dimeric assemblies were constructed in Avogadro to generate the supramolecular arrangements corresponding to **(*S*)-AggI** and **(*S*)-AggII**. Distance constraints were applied to the BF₄⁻ counterions to prevent ion pairing with the Pd center in hexane, yielding **(*S*)-AggI**. The alternative packing mode **(*S*)-AggII** was obtained by laterally displacing the counterions by approximately 1 Å relative to the Pd center followed by constrained geometry reoptimization under the same conditions.

Higher-order oligomers ranging from dimers to decamers were subsequently constructed from the optimized geometries while preserving the relative orientation of the monomeric units and the directionality of the hydrogen-bonding interactions. All optimized structures were subsequently visualized using PyMOL 3.1.4 for accurate depiction of the spatial arrangement.

**Binding Energy Analysis and Structural Characterization**

To gain deeper insights into the geometric features and cooperative growth of the supramolecular assemblies, decamer models of **(*S*)-AggI** and **(*S*)-AggII** were constructed from the optimized oligomer geometries and further optimized at the GFN2-xTB level following literature-reported methodologies.^[13–15]^

To minimize terminal effects, external monomer units were sequentially removed from the decamer stacks to generate a series of shorter oligomers (n = 10, 8, 6, 4, 2). Binding energies were then evaluated through single-point energy calculations, and the binding energy per added monomer pair (*ΔE_bind, n−1_*) was determined according to:

$${\Delta E}_{bind,n-1}=\frac{E_{Stack}-n*E_{monomer}}{n-1}$$

For both conformers, *ΔE_bind,n−1_* values displayed an exponential decay with increasing oligomer length, indicative of a cooperative supramolecular polymerization process (Figures S42 and S43), in agreement with the self-assembly mechanisms previously proposed for **(*S*)-AggI** and **(*S*)-AggII**, respectively (Figure 2e and 2f of the main article).

Key structural parameters were extracted from the optimized oligomer geometries to compare the packing characteristics of **(*S*)-AggI** and **(*S*)-AggII**. In particular, pyridine coplanarity was quantified using the dihedral angle *ϕ*, defined as the torsion describing the relative orientation of the pyridine rings within the monomer unit.

Additionally, the torsional angle *θ* between adjacent monomer units was measured to evaluate the degree of helical distortion along the supramolecular stack. These parameters were obtained from the optimized oligomer geometries using Avogadro 1.2.0.

Average values were calculated across the stacks to provide representative structural parameters for each aggregate. The analysis reveals clear differences between the two packing modes: **(*S*)-AggI** displays reduced pyridine coplanarity and larger torsional distortions, whereas **(*S*)-AggII** adopts a more planar arrangement consistent with the formation of extended supramolecular fibers observed experimentally.

**Circular Dichroism (CD) Simulations**

To confirm the chiral arrangement of **(*S*)-AggII**, ECD spectra were calculated for a hexamer model of **(*S*)-AggII**. Excited-state properties were computed using the simplified Tamm–Dancoff approximation (sTDA) combined with GFN2-xTB,^[9.10]^ specifically via the xtb4stda interface. An energy window of 10 eV was applied to ensure accurate coverage of relevant transitions.

The raw data were processed using SpecDis 1.71,^[16]^ applying a Gaussian broadening function (0.16 eV) to simulate the final spectra. The computed CD profiles for **(*S*)-AggI** and **(*S*)-AggII** are in good agreement with the experimental signatures (Figure S42 and S43), supporting the proposed helical supramolecular stacking.

**Figure S36.** Minimum-energy structures of **(*S*)-AggI** oligomers computed at the GFN2-xTB level: a) dimer (2), b) trimer (3), c) tetramer (4), and d) pentamer (5).

**Figure S37.** Minimum-energy structures of **(*S*)-AggI** oligomers computed at the GFN2-xTB level: a) hexamer (6), b) heptamer (7) and c) octamer (8).

**Figure S38.** Minimum-energy structures of **(*S*)-AggII** oligomers computed at the GFN2-xTB level: a) dimer (2), b) trimer (3), c) tetramer (4), and d) pentamer (5).

**Figure S39.** Minimum-energy structures of **(*S*)-AggII** oligomers computed at the GFN2-xTB level: a) hexamer (6), b) heptamer (7) and c) octamer (8).

**Figure S40.** a) Front view of the optimized 10-mer stack of **(*S*)-AggI** at the GFN2-xTB level. b) Binding energies of oligomers (n = 2–10) derived by sequentially removing terminal units from the optimized 10-mer, calculated via single-point energy calculations. c) Comparison between the experimental CD spectrum of **(*S*)-AggI** in MCH and the simulated CD spectrum of a **(*S*)-AggI** hexamer obtained using sTDA-xTB

**Table S4.** Stacking rotation angles (*θ)* and average intermolecular distances between adjacent molecules in the 10-mer of **(*S*)-AggI**, computed at the GFN2-xTB level.

| **Monomeric units** | **Rotation angle**  ***θ* (deg)** | **Pd⋅⋅⋅Pd distance**  **(Å)** | **Average. H-bond**  **distance (Å)** |  |
| --- | --- | --- | --- | --- |
| 1^st^ and 2^nd^ | -9.5 | 5.132 | 2.518 |  |
| 2^nd^ and 3^rd^ | -15.1 | 5.002 | 2.581 |  |
| 3^rd^ and 4^th^ | -15.2 | 5.004 | 2.591 |  |
| 4^th^ and 5^th^ | -15.3 | 5.004 | 2.591 |  |
| 5^th^ and 6^th^ | -15.8 | 5.003 | 2.592 |  |
| 6^th^ and 7^th^ | -15.6 | 5.001 | 2.592 |  |
| 7^th^ and 8^th^ | -15.8 | 5.004 | 2.591 |  |
| 8^th^ and 9^th^ | -15.6 | 4.999 | 2.576 |  |
| 9^th^ and 10^th^ | -9.5 | 5.136 | 2.491 |  |
| Total average | -14.2 | 5.032 | 2.569 |  |

**Table S5.** Pyridine–pyridine dihedral angles (*ϕ)* and average B···Pd distances between adjacent molecules in the 10-mer of **(*S*)-AggI**, computed at the GFN2-xTB level.

| **Monomeric units** | **Dihedral angle Py-Pd-Py**  ***ϕ* (deg)** | **Average B⋅⋅⋅Pd**  **Distance (Å)** |  |
| --- | --- | --- | --- |
| 1 | -23.0 | 4.747 |  |
| 2 | -39.4 | 4.778 |  |
| 3 | -38.2 | 4.775 |  |
| 4 | -38.3 | 4.772 |  |
| 5 | -38.3 | 4.773 |  |
| 6 | -38.4 | 4.773 |  |
| 7 | -38.3 | 4.777 |  |
| 8 | -38.3 | 4.776 |  |
| 9 | -38.9 | 4.779 |  |
| 10 | -24.8 | 4.769 |  |
| Total average | -35.6 | 4.772 |  |

**Figure S41.** a) Front view of the optimized 10-mer stack of **(*S*)-AggII** at the GFN2-xTB level. b) Binding energies of oligomers (n = 2–10) derived by sequentially removing terminal units from the optimized 10-mer, calculated via single-point energy calculations. c) Comparison between the experimental CD spectrum of **(*S*)-AggI** in MCH and the simulated CD spectrum of a **(*S*)-AggII** hexamer obtained using sTDA-xTB

**Table S6.** Stacking rotation angles (*θ)* and average intermolecular distances between adjacent molecules in the 10-mer of **(*S*)-AggII**, computed at the GFN2-xTB level.

| **Monomeric units** | **Rotation angle**  ***θ* (deg)** | **Pd⋅⋅⋅Pd distance**  **(Å)** | **Average. H-bond**  **distance (Å)** |
| --- | --- | --- | --- |
| 1^st^ and 2^nd^ | 7.0 | 5.511 | 1.984 |
| 2^nd^ and 3^rd^ | 7.5 | 4.863 | 1.981 |
| 3^rd^ and 4^th^ | 7.0 | 4.823 | 1.993 |
| 4^th^ and 5^th^ | 6.8 | 4.846 | 2.005 |
| 5^th^ and 6^th^ | 6.5 | 5.145 | 2.016 |
| 6^th^ and 7^th^ | 7.5 | 4.865 | 2.024 |
| 7^th^ and 8^th^ | 7.2 | 4.852 | 2.000 |
| 8^th^ and 9^th^ | 7.1 | 4.896 | 2.052 |
| 9^th^ and 10^th^ | 7.5 | 5.154 | 1.997 |
| Total average | 7.1 | 4.995 | 2.007 |

**Table S7.** Pyridine–pyridine dihedral angles (*ϕ)* and average B···Pd distances between adjacent molecules in the 10-mer of **(*S*)-AggII**, computed at the GFN2-xTB level.

| **Monomeric units** | **Dihedral angle Py-Pd-Py**  ***ϕ* (deg)** | **Average B⋅⋅⋅Pd**  **Distance (Å)** |  |
| --- | --- | --- | --- |
| 1 | -66.3 | 5.598 |  |
| 2 | -70.1 | 5.593 |  |
| 3 | -63.7 | 5.612 |  |
| 4 | -68.3 | 5.629 |  |
| 5 | -66.1 | 5.576 |  |
| 6 | -65.7 | 5.617 |  |
| 7 | -63.1 | 5.622 |  |
| 8 | -63.4 | 5.625 |  |
| 9 | -69.7 | 5.596 |  |
| 10 | -66.6 | 5.579 |  |
| Total average | -66.3 | 5.604 |  |

*Influence of counterion on asymmetry amplification.*

Figure S42. CD absorption spectra of MR experiments between a) (*S*)-AggI (blue solid line) and (*R*)-AggI (blue dashed line), as well as between b) (*S*)-AggII (green solid line) and (*R*)-AggII (green dashed line). c) Changes of CD intensity in *MR* experiments of AggI (top) and AggII (bottom) as a function of *ee* values. *ee* = 1.0 corresponds to pure (*S*)-AggI or (*S*)-AggII, whereas *ee* = −1.0 corresponds to pure (*R*)-AggI and (*R*)-AggII. CD spectra of *SaS* experiments between d) (*S*)-AggI (blue solid line) and 2-AggI (light green solid line), as well as between e) (*S*)-AggII (dark green solid line) and 2-AggII (light green solid line). f) Changes in CD intensity in *SaS* experiments for AggI (top) and AggII (bottom) as a function of sergeants’ fraction. The red lines in (c) and (f) were set to guide the eye. (*c* = 50 µM, *T* = 298 K in MCH:DCM 8:2)

After analyzing the role of the counterion in governing the helicity of (*S*)-AggI and (*S*)-AggII, we ultimately investigated how these structurally different assemblies influence chirality amplification within the system. We first explored majority rules (*MR*) effects by mixing solutions of the kinetically controlled aggregates (*S*)-AggI and (*R*)-AggI at varying ratios in MCH:DCM 8:2 at 298 K maintaining a total concentration of 50 μM. The CD spectra show a gradual evolution of the CD signal as the molar ratio of (*S*)-1 and (*R*)-1 changes, with zero-crossing points near 335 nm and 305 nm (Figure S42a). However, the changes in spectral intensity indicate only a limited degree of an ampliﬁcation effect. To quantify this behavior, the CD intensity at 332 nm was plotted against the enantiomeric excess (Figure S42c, top), yielding a shallow sigmoidal curve. Maximum chiral amplification was only achieved at approximately 60% *ee*, indicating a weak *MR* effect for AggI. On the other hand, the thermodynamically favored aggregate (AggII) exhibited a slightly more pronounced *MR* effect under identical conditions. The CD spectra (Figure S42b) show a pronounced increase in signal intensity with increasing *ee*, together with well-defined bisignate features and zero-crossing points at 348 nm and 304 nm. Plotting the CD intensity at 332 nm against *ee* (Figure S42c, bottom) results in a sigmoidal curve, indicative of chiral amplification. The maximum response is observed at around 50% *ee*, slightly lower than that of AggI (≈60% *ee*), suggesting more efficient chirality transfer in the thermodynamic aggregate. Notably, the UV–vis spectra remain essentially unchanged upon variation of the *ee* (Figure S43), indicating that these differences arise from changes in supramolecular chirality rather than variations in aggregation state. Although the difference between the two systems is moderate, the slightly enhanced amplification effect observed for AggII may be related to its one-dimensional morphology, which likely facilitates more effective propagation of chiral information compared to the nanoparticle assemblies of AggI.

To further evaluate the amplification of asymmetry within the assemblies, the sergeants-and-soldiers (*SaS*) principle was examined using (*S*)-1 or (*R*)-1 as chiral sergeants and achiral 2 as the soldier. Co-assemblies were prepared at different molar ratios while maintaining a constant total concentration (*c*_T_ = 50 μM). For the kinetically controlled state, gradual addition of (*S*)-AggI to solutions of 2 in MCH:DCM 8:2 at 298 K (2-AggI) results in a linear increase of the characteristic CD signal (Figure S42d). Plotting the CD intensity at 332 nm against the molar fraction of (*S*)-AggI yields a nearly linear dependence, indicating negligible asymmetry amplification, likely due to limited structural communication between the two components. In contrast, *SaS* experiments involving the thermodynamic aggregates (*S*)-AggII and 2-AggII display a weakly nonlinear dependence with a maximum response at ca. 60% sergeant content, indicating modest amplification of asymmetry.

To quantify these effects, the mismatch penalty (*MMP*) and the helical reversal penalty (*HRP*) were determined from the CD data for both aggregated states (Figures S44-S46).^[17–19]^ AggI exhibits an *MMP* of –6.27 kJ mol^-1^ and an *HRP* of –9.23 kJ mol^-1^, indicating that a relatively large enantiomeric bias is required to achieve full helicity inversion.^[20]^ In comparison, AggII shows an *MMP* of –5.12 kJ mol^-1^ and an *HRP* of –10.12 kJ mol^-1^, consistent with a slightly more robust and cooperative helical structure. Overall, these results demonstrate that differences in supramolecular morphology significantly influence the efficiency of chiral amplification, highlighting how counterion-dependent assembly can be exploited to modulate helicity control in supramolecular polymers.

**Figure S43.** UV-vis spectra of *MR* of: a) **(*S*)-AggI** (dark blue), **(*R*)-AggI** (blue dashed line); c) **(*S*)-AggII** (green), **(*R*)-AggII** (green dashed line). UV-vis spectra of *SaS* of: b) **(*S*)-AggI** (blue), **2-AggI** (blue dashed line); b) **(*S*)-AggII** (green), **2-AggII** (green dashed line).

**Figure S44.** a) Simultaneous fit on both data sets at the minimum. Based on the fitting results, the *HRP* value was determined to be -9.23 kJ mol^-1^. b) Contour plots of the sum of squared residuals by fitting *SaS* data in both supramolecular copolymer **AggI** with the minimum at which the dotted lines cross (*σ* = 1× 10^–3^ and *ω* = 0.701). c) Simultaneous fit on both data sets at the minimum. Based on the fitting results, the *HRP* value was determined to be -10.12 kJ mol^-1^. d) Contour plots of the sum of squared residuals by fitting *SaS* data in both data in both supramolecular copolymer **AggII** with the minimum at which the dotted lines cross (*σ* = 2.47× 10^–4^ and *ω* = 0.879).

**Figure S45**. a) Fit on the data set at the minimum in **(*S*)-1** compound excess. b) Contour plot of the sum of squared residuals obtained by fitting *MR* data in both supramolecular copolymer **AggI** data with the minimum at which the dotted lines cross (*σ* = 1.68× 10^–5^ and *ω* = 0.233). c) Fit on the data set at the minimum in **(*R*)** compound excess. d) Contour plot of the sum of squared residuals obtained by fitting *MR* data in both supramolecular copolymers of **Agg I** data with the minimum at which the dotted lines cross (*σ* = 8.57× 10^–6^ and *ω* = 0.158). Averaged *MMP* = -6.27 kJ/mol.

**Figure S46.** a) Fit on the data set at the minimum in **(*S*)-1** compound excess. b) Contour plot of the sum of squared residuals obtained by fitting *MR* data in both supramolecular copolymer **AggII** data with the minimum at which the dotted lines cross (*σ* = 4.48× 10^–6^ and *ω* = 0.042). c) Fit on the data set at the minimum in **(*R*)** compound excess. d) Contour plot of the sum of squared residuals obtained by fitting *MR* data in both supramolecular copolymers of **Agg II** data with the minimum at which the dotted lines cross (*σ* = 8.97× 10^–4^ and *ω* = 0.963). Averaged *MMP* = -5.12 kJ/mol.

## **References**

[1]	Q. D. Wang, S. X. Zhang, Z. W. Zhang, Y. Wang, M. Ma, X. Q. Chu, Z. L. Shen, “Palladium-Catalyzed Sonogashira Coupling of a Heterocyclic Phosphonium Salt with a Terminal Alkyne” *Org. Lett.* **2022**, *24*, 4919–4924.

[2]	A. T. Wright, J. D. Song, B. F. Cravatt, “A suite of activity-based probes for human cytochrome P450 enzymes” *J. Am. Chem. Soc.* **2009**, *131*, 10692–10700.

[3]	L. Holzhauer, C. Liagre, O. Fuhr, N. Jung, S. Bräse, “Scope of tetrazolo[1,5-a]quinoxalines in CuAAC reactions for the synthesis of triazoloquinoxalines, imidazoloquinoxalines, and rhenium complexes thereof” *Beilstein J. Org. Chem.* **2022**, *18*, 1088–1099.

[4]	P. A. Korevaar, C. Grenier, A. J. Markvoort, A. P. H. J. Schenning, T. F. A. De Greef, E. W. Meijer, “Model-driven optimization of multicomponent self-assembly processes” *Proc. Natl. Acad. Sci. U. S. A.* **2013**, *110*, 17205–17210.

[5]	G. Meisl, J. B. Kirkegaard, P. Arosio, T. C. T. Michaels, M. Vendruscolo, C. M. Dobson, S. Linse, T. P. J. Knowles, “Molecular mechanisms of protein aggregation from global fitting of kinetic models” *Nat. Protoc.* **2016**, *11*, 252–272.[59]

[6]	N. Bäumer, K. K. Kartha, S. Buss, I. Maisuls, J. P. Palakkal, C. A. Strassert, G. Fernández, “Tuning energy landscapes and metal-metal interactions in supramolecular polymers regulated by coordination geometry” *Chem. Sci*. **2021**, 12, 5236–5245.

[7]	N. Bäumer, K. K. Kartha, S. Buss, J. P. Palakkal, C. A. Strassert, G. Fernández, “Exploiting coordination geometry to tune the dimensions and processability of metallosupramolecular polymers” *Org. Chem. Front*. **2021**, 8, 4138–4143.

[8]	M. D. Hanwell, D. E. Curtis, D. C. Lonie, T. Vandermeersch, E. Zurek, G. R. Hutchison, *“Avogadro: an advanced semantic chemical editor, visualization, and analysis platform”*, *J. Cheminform*. **2012**, *4*,1-17.

[9]	S. Grimme, C. Bannwarth, “Ultra-fast computation of electronic spectra for large systems by tight-binding based simplified Tamm-Dancoff approximation (sTDA-xTB)” *J. Chem. Phys.* **2016**, *145*, 054103.

[10]	C. Bannwarth, S. Ehlert, S. Grimme, “GFN2-xTB - An Accurate and Broadly Parametrized Self-Consistent Tight-Binding Quantum Chemical Method with Multipole Electrostatics and Density-Dependent Dispersion Contributions” *J. Chem. Theory Comput.* **2019**, *15*, 1652–1671.

[11]	A. De León, J. Pons, X. Solans, M. Font-Bardia, “Tetrakis(pyridine-kN)palladium(II) bis-(tetrafluoridoborate)” *Acta Crystallogr. Sect. E* **2007**, *63*, m2164.

[12]	G. Kurpik, A. Walczak, M. Gołdyn, J. Harrowfield, A. R. Stefankiewicz, “Pd(II) Complexes with Pyridine Ligands: Substituent Effects on the NMR Data, Crystal Structures, and Catalytic Activity” *Inorg. Chem.* **2022**, *61*, 14019–14029.

[13]	C. Montañez-Moyano, Y. Xue, M. V. Cappellari, A. Martínez-Manjarrés, L. Borsdorf, C. A. Strassert, G. Fernandez, “Controlling Asymmetry Amplification through Supramolecular Polymorphism” *Angew. Chem. Int. Ed.* **2025**, *64*, e202509031.

[14]	M. Lago-Silva, M. M. Cid, E. Quiñoá, F. Freire, “Dynamic Axial-to-Helical Communication Mechanism in Poly[(allenylethynylenephenylene)acetylene]s under External Stimuli” *Angew. Chem. Int. Ed.* **2023**, *62*, e202303329.

[15]	E. E. Greciano, R. Rodríguez, K. Maeda, L. Sánchez, “Disclosing chirality in consecutive supramolecular polymerizations: Chiral induction by light in: N-annulated perylenetetracarboxamides” *Chem. Commun.***2020**, *56*, 2244–2247.

[16]	T. Bruhn, A. Schaumlöffel, Y. Hemberger, G. Bringmann, “SpecDis: Quantifying the comparison of calculated and experimental electronic circular dichroism spectra” *Chirality* **2013**, *25*, 243–249.

[17]	Y. Xue, C. Zhang, T. Lv, L. Qiu, F. Wang, “Amplification of Dissymmetry for Circularly Polarized Photodetection by Cooperative Supramolecular Polymerization” *Angew. Chem. Int. Ed.* **2023**, *62*, e202300972.

[18]	F. Helmich, M. M. J. Smulders, C. C. Lee, A. P. H. J. Schenning, E. W. Meijer, “Effect of stereogenic centers on the self-sorting, depolymerization, and atropisomerization kinetics of porphyrin-based aggregates” *J. Am. Chem. Soc.* **2011**, *133*, 12238–12246.

[19]	M. M. J. Smulders, P. J. M. Stals, T. Mes, T. F. E. Paffen, A. P. H. J. Schenning, A. R. A. Palmans, E. W. Meijer, “Probing the Limits of the Majority-Rules Principle in a Dynamic Supramolecular Polymer” *J. Am. Chem. Soc.* **2010**, *132*, 620–626.

[20]	E. E. Greciano, J. Calbo, J. Buendía, J. Cerdá, J. Aragó, E. Ortí, L. Sánchez, “Decoding the Consequences of Increasing the Size of Self-Assembling Tricarboxamides on Chiral Amplification” *J. Am. Chem. Soc.* **2019**, *141*, 7463–7472.
